# SARS-CoV-2 evolution on a dynamic immune landscape

**DOI:** 10.1038/s41586-024-08477-8

**Published:** 2025-01-29

**Authors:** N. Alexia Raharinirina, Nils Gubela, Daniela Börnigen, Maureen Rebecca Smith, Djin-Ye Oh, Matthias Budt, Christin Mache, Claudia Schillings, Stephan Fuchs, Ralf Dürrwald, Thorsten Wolff, Martin Hölzer, Sofia Paraskevopoulou, Max von Kleist

**Affiliations:** 1 Department of Mathematics & Computer Science, Freie Universität Berlin, Berlin, Germany; 2 International Max-Planck Research School for Biology and Computation (IMPRS-BAC), Max-Planck Institute for Molecular Genetics, Berlin, Germany; 3 Project Groups, Robert Koch Institute, Berlin, Germany; 4 Department 1, Robert Koch Institute, Berlin, Germany; 5 Department MFI, Robert Koch Institute, Berlin, Germany

**Keywords:** Epidemiology, Data integration, Viral infection, Viral infection

## Abstract

Since the onset of the pandemic, many SARS-CoV-2 variants have emerged, exhibiting substantial evolution in the virus’ spike protein^1^, the main target of neutralizing antibodies^2^. A plausible hypothesis proposes that the virus evolves to evade antibody-mediated neutralization (vaccine- or infection-induced) to maximize its ability to infect an immunologically experienced population^1,3^. Because viral infection induces neutralizing antibodies, viral evolution may thus navigate on a dynamic immune landscape that is shaped by local infection history. Here we developed a comprehensive mechanistic model, incorporating deep mutational scanning data^4,5^, antibody pharmacokinetics and regional genomic surveillance data, to predict the variant-specific relative number of susceptible individuals over time. We show that this quantity precisely matched historical variant dynamics, predicted future variant dynamics and explained global differences in variant dynamics. Our work strongly suggests that the ongoing pandemic continues to shape variant-specific population immunity, which determines a variant’s ability to transmit, thus defining variant fitness. The model can be applied to any region by utilizing local genomic surveillance data, allows contextualizing risk assessment of variants and provides information for vaccine design.

## Abstract (web summary)

A modelling approach predicts SARS-CoV-2 variant dynamics on the basis of immunity and cross-neutralization, which was shaped by a region’s SARS-CoV-2 infection history.

## Main

SARS-CoV-2 is still spreading at high rates^6^, with thousands of patients in intensive care units and reported fatalities monthly^7^, notwithstanding long-term effects^8^. Although approximately 775 million cases have been reported so far^9^, true numbers are magnitudes larger, and it is reasonable to assume that almost the entire world population has been exposed to viral antigen so far^10^.

Since the onset of the pandemic, SARS-CoV-2 has evolved substantially in the spike protein^1^, which harbours all epitope sites for neutralizing antibodies elicited by vaccines or infection^2^. For example, the Omicron lineages BA.1 and BA.2 contained multiple alterations in spike epitopes, allowing them to infect vaccinated and convalescent individuals^11–14^, who represented most of the population at the time. Although inter-country differences in viral evolution existed early in the pandemic^15^, the geographical variation of emerging Omicron sublineages reflects an increasing complexity of the global immunological landscape, in which the course of infection waves with new (sub)variants in a particular region could be substantially influenced by the infection history of that region (that is, which variants dominated the preceding waves and at what time). This circumstance poses challenges in optimizing the design of adapted mRNA vaccines to protect vulnerable groups or heavily exposed individuals. Despite the appreciation of the problem^16^ and the abundance of rich data sources for SARS-CoV-2, there has been limited progress in integrating the available data to inform variant risk assessment and vaccine design.

**Fig. 1:**
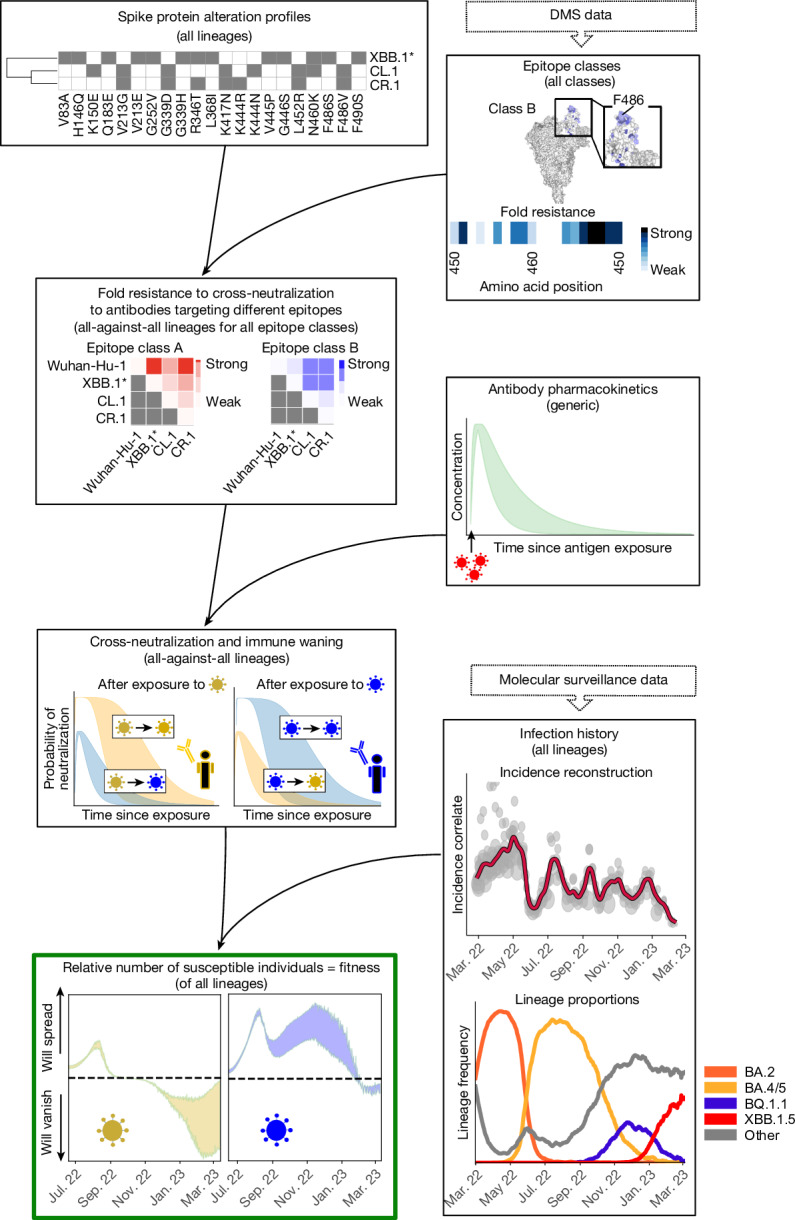
Overview of data sources and data integration for modelling variant-specific immunity and fitness advantage. DMS data are used to define fold resistance to cross-neutralization (FR; that is, factor of decreased antibody potency), conferred by mutational differences between lineages at amino acid positions relevant to antibodies of an epitope class (top right). This information is combined with the mutational profiles of the distinct lineages (top panel) to compute ‘fold resistance maps’ to cross-neutralization between any pair of lineages (*x*, *y*) for each epitope class (left, second panel from top). Asterisks (for example, XBB.1*) indicate spike pseudo groups; that is, the group of lineages with identical mutations in the spike protein (for example, identical to XBB.1, in the case of XBB.1*). Antibody pharmacokinetics (right, second panel from the top) after antigen exposure are then combined with the fold resistance maps to compute the temporal profiles of virus cross-neutralization between any pair (*x*, *y*) of immunity-inducing variant *x* and another variant *y*. Finally, these variant-resolved immune waning dynamics are combined with the timeline of infection (lower right panels) with distinct variants in any region of interest, to compute the relative number of susceptible individuals *γ*_*y*_(*t*) (lower left) for each variant over time. The relative number of susceptible individuals *γ*_*y*_(*t*) indicates the competitive fitness advantage of each variant: if *γ*_*y*_(*t*) > 0, variant *y* has the potential to spread, and if *γ*_*y*_(*t*) < 0, the variant will decrease in frequency and eventually vanish.

In this work, we reason that SARS-CoV-2 evolution is driven by infection history and the ability of elicited humoral immunity to cross-neutralize emerging variants. To this end, we conjecture that the variant-specific relative number of susceptible individuals predicts the relative fitness (and evolution) of SARS-CoV-2 in a region of interest, over time. To calculate this quantity, we integrated available data as summarized in Fig. 1: deep mutational scanning (DMS) data^5,17^ identified epitopes of neutralizing (spike-targeting) antibodies, which, when combined with lineage-specific spike alteration profiles, allowed us to compute cross-neutralization between any pair of variants. By integrating antibody pharmacokinetics, we could then compute how much and how long a recovery from a recent infection with a variant will protect against another variant. Last, we reconstructed the infection timeline in the region of interest and combined this timeline with historical SARS-CoV-2 variant dynamics to estimate the expected number of susceptible individuals for each variant. If SARS-CoV-2 evolution was decisively driven by population immunity, the variant-specific number of susceptible individuals should be directly proportional to the variant-specific transmission rate, and hence our model would allow us to estimate the competitive growth (dis-) advantage (that is, the relative fitness) of any given variant.

After introducing the main methodological concepts, we will first demonstrate our analytical approach using data from the German COVID-19 epidemic, followed by an application to international datasets. Finally, we will highlight regional differences in the immune landscape and their impact on variant dynamics.

## Utilization of DMS data

**Fig. 2:**
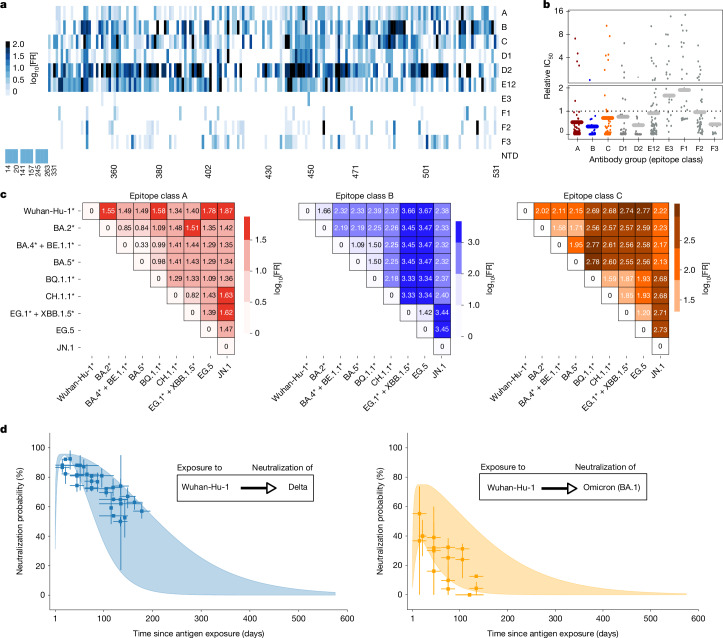
Cross-neutralization and immune waning dynamics. **a**, Fold resistance induced by mutational differences against antibodies of distinct epitope classes at indicated sites. **b**, Relative potency IC_50(DMS)_ of antibodies of specific epitope classes in the DMS data. The solid horizontal lines show the respective average IC_50(DMS)_. The dotted horizontal line marks the average across all epitope classes. **c**, Fold resistance to neutralization against immunity-inducing variants (on *y* axis) for antibodies of epitope classes A, B and C. Asterisks indicate spike pseudo groups; that is, lineages with identical mutations in the spike protein. **d**, Predicted neutralization probability of the Delta variant (blue range, left panel) and the Omicron variant (BA.1) (orange range, right panel) after exposure to the Wuhan-Hu-1 antigen *P*_Neut_(*t*, Wuhan-Hu-1, Delta). Ranges depict minimum–maximum ranges resulting from pharmacokinetic variability. The corresponding clinical vaccine efficacy is also indicated (blue and orange markers, central estimate; vertical line, 95% confidence interval; horizontal line, range of time post vaccination as stated in the original study; see Supplementary Table 5). Source Data

**Extended Data Fig. 1:**
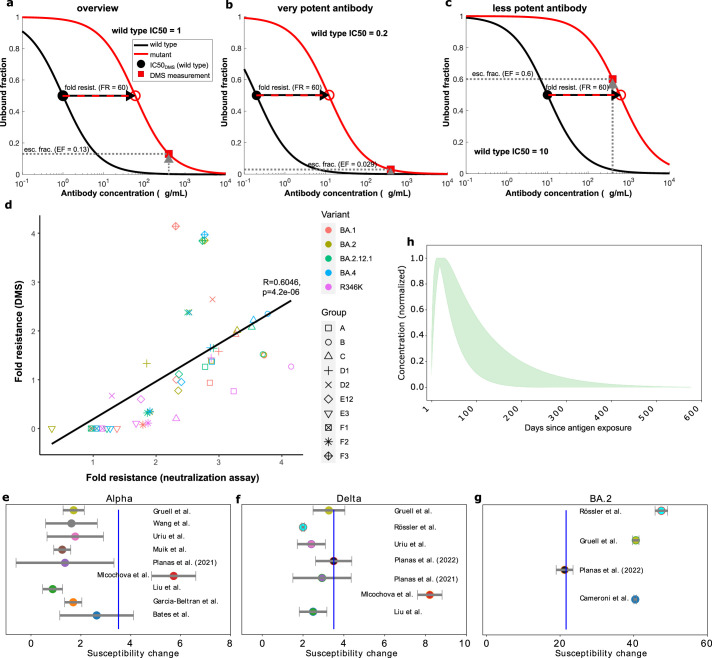
Utilization of DMS data and comparison of DMS-derived fold resistances with titer changes from mono- and polyclonal sera. **a**. Theoretical binding curve of an antibody *a* to a wild-type epitope of SARS-CoV-2 (black line) and corresponding binding curve to an epitope that is mutated at site *s* (red line). The black dot and red circle mark the concentrations (x-axis) where the binding is half maximal (y-axis) for the wild type *IC50*_*DMS*_*(a)* and mutant virus *IC50*_*DMS*_*(a,s)* respectively. The ‘fold resistance’ (red-black dashed line) denotes the shift in the *IC50*, such that *IC50*_*DMS*_*(a,s)* = *FR(a, s) · IC50*_*DMS*_*(a)*. The red square marks the DMS-measured unbound fraction (escape fraction *ef(s, a)*) and the upward-pointing grey arrow the concentration at which the DMS experiment was conducted. Both, *IC50*_*DMS*_*(a)*, *ef(s, a)* were measured in the original DMS experiment at an antibody concentration of 400 *μg/mL*. However, the same DMS experiment, performed with a more- (panel **b**.) or less (panel **c**.) potent antibody would yield a smaller, respectively bigger escape fraction, while the phenotypic effect *FR(a, s)* of the mutation s is quantitatively identical. To avoid these artifacts, we calculated *FR(a, s)* from the original DMS data. **d**. Comparison between DMS-derived fold resistances *FR*_*x,y*_ and fold resistances derived from neutralization assays (mono-clonal). Distinct markers show the epitope classes and distinct colors the Spike-pseudo-groups. **e-g**. Comparison between DMS-derived changes in neutralization and neutralization titer changes (polyclonal sera). Geometric mean change in neutralization titers for Alpha (**e**.), Delta (**f**.) and BA.2 (**g**.) after exposure to the Wuhan antigen are highlighted by dots (± standard deviation), whereas DMS-based model predictions are shown as a blue vertical line and were computed as outlined in^17,62^. Raw data on neutralization titer changes^21,63–73^. **h**. Normalized antibody pharmacokinetics after antigen exposure. Parameters and equations given in the Methods section.

We utilized DMS data to compute cross-neutralization profiles for each possible pair of ‘antibody-inducing’ variant *x* and cross-neutralized variant *y* (upper panels in Fig. 1). In the original DMS data, ‘escape fractions’ were assigned to all sites in the receptor-binding domain (RBD) region for a large panel of 836 antibodies^4,5,17^, which were aggregated into 10 epitope classes^18^ as highlighted in Fig. 2a,b (details on antibodies and classes in Supplementary Tables 1 and 2). We processed the original DMS data to derive ‘fold resistances’ to cross-neutralization (Extended Data Fig. 1a–c) between antibody-inducing and antigen-presenting lineages (Fig. 2a and Methods). Intuitively, fold resistance values denote how much more antibody is needed to neutralize mutant virus *y* to the same extent as antibody-inducing lineage *x*. A comparison between fold resistances obtained through our method from DMS data with fold resistances obtained through neutralization assays yielded overall good agreement for monoclonal sera (Extended Data Fig. 1d), as well as polyclonal sera (Extended Data Fig. 1e–g). Next, we computed a fold resistance for each epitope class and for each pair of antibody-inducing lineage *x* and cross-neutralized lineage *y* on the basis of their genetic profiles and the DMS data. Fold resistances for three representative epitope classes and a set of relevant Omicron lineages are shown in Fig. 2c.

## Protection against viral infection

Although cross-neutralization potency can qualitatively describe the antigenic overlap between variants, the ability to neutralize the virus (and prevent infection) depends on the neutralizing antibody concentrations at the time of viral re-exposure^19^.

Neutralizing antibody levels rise within 1 to 2 weeks after initial antigen exposure and slowly decay afterwards. We parameterized a simple pharmacokinetic model to capture the antibody concentration–time profile after initial antigen exposure (Methods and Extended Data Fig. 1h). Then, on the basis of DMS data, we computed a relative weighting of antibodies belonging to different epitope classes, as shown in Fig. 2b. This data-derived weighting may reflect the accessibility of the distinct epitopes, the strength of binding or the capability to neutralize the virus when an antibody is bound. Our analysis showed that antibodies of epitope classes A, B, D2 and F3 more potently neutralize the virus, whereas antibodies of classes E3 and F1 are less potent.

On the basis of the previously computed cross-neutralization potency, antibody pharmacokinetics and relative antibody potencies, we subsequently estimated the probability of neutralizing the Delta variant (genetic profile in Supplementary Table 3) after exposure to the Wuhan-Hu-1-variant antigen (Fig. 2d; data sources in Supplementary Table 4). Assuming that neutralization probability approximates infection risk reduction, we calibrated the model by estimating its only free parameter (the average normalized half-maximal inhibitory antibody concentration $\widehat{\,{{\rm{IC}}}_{{50}_{(x)}}}$). After this step, no further model calibration was performed.

Notably, utilized clinical data vary considerably in the level of reported risk reduction owing to statistical limitations (few observed ‘infection events’), as well as heterogeneity in the study groups, which is a well-known phenomenon for prevention trials^20^. Regarding immune waning dynamics after exposure to the Wuhan-Hu-1-variant antigen, we observe that Delta can be almost completely neutralized shortly after antigen exposure. However, neutralization probabilities decrease to 50%, depending on individual antibody pharmacokinetics, within 100 to 250 days after exposure to the Wuhan-Hu-1-variant antigen (Fig. 2d).

We then applied the calibrated model to predict Omicron (BA.1) neutralization after exposure to the Wuhan-Hu-1-variant antigen. Our predictions align well with booster-vaccine efficacy data (summarized in Supplementary Table 5): our model predicts that Omicron BA.1 (genetic profile in Supplementary Table 3) is initially neutralized with 45–85% probability approximately 2 weeks after exposure to the Wuhan-Hu-1-variant antigen, with neutralizing immunity decaying rapidly and reaching about 10% infection risk reduction between 80 and 350 days post antigen exposure. Note that these predictions are also broadly in agreement with in vitro neutralization data^21^.

## Immune and variant dynamics in Germany

**Extended Data Fig. 2:**
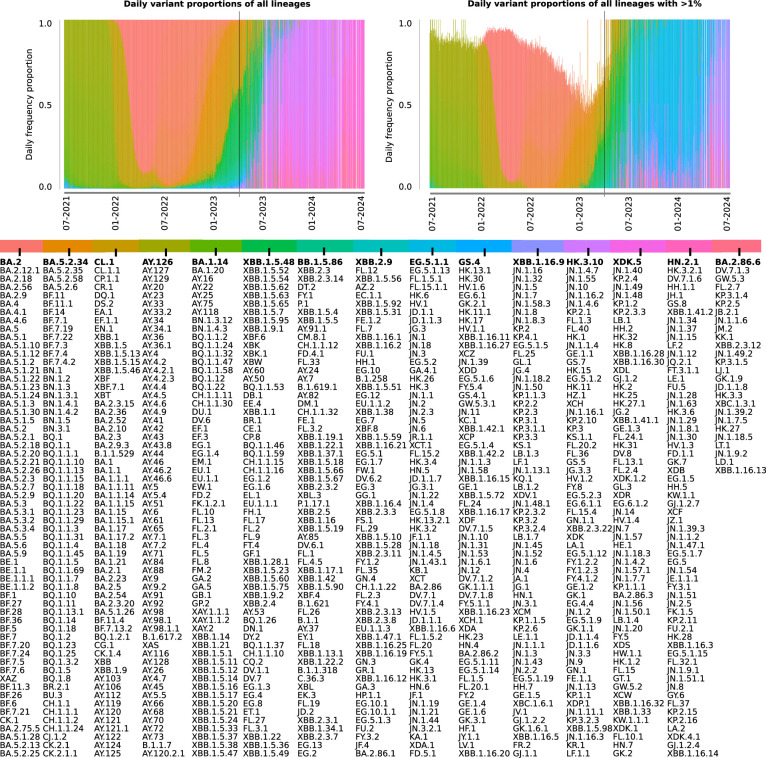
Spike-pseudo-group dynamics in Germany over the investigated time horizon. For readability we only plot pseudo-groups (groups of lineages with identical mutation profiles in the spike protein) that appear at a frequency of > 1%.

Next, we utilized the developed model to estimate the immune landscape in Germany and assess whether it predicts variant dynamics. To apply the model to Germany, we integrated data from the national virus genomics surveillance, selecting randomly collected virus genomes between 1 July 2021 and 1 July 2024 (≈607,000 sequences; Supplementary Table 6) to predict immune dynamics between 1 March 2022 and 1 July 2024. The time from 1 July 2021 to 1 March 2022 served as a ‘burn-in’ phase to converge to the initial immunological landscape. The viral genomes from the German dataset belonged to 1,718 Pangolin lineages and harboured 124 and 219 alterations in the RBD and amino-terminal domain (NTD), respectively. Of these initial 1,718 lineages, 756 had unique spike profiles (‘spike pseudo-groups’). A total of 227 spike pseudo-groups occurred at a frequency greater than 1% on at least 1 calendar day (representing 1,098 pangolin lineages; Extended Data Fig. 2).

We reconstructed the timeline of infection, and subsequently estimated the variant-specific infection timeline on the basis of lineage frequencies. The variant-specific timeline of infection was then incorporated into the DMS-derived cross-neutralization and immune waning model (previous paragraphs) to reconstruct the immunological landscape in Germany (Fig. 1 (lower panels) and Methods).

**Extended Data Fig. 3:**
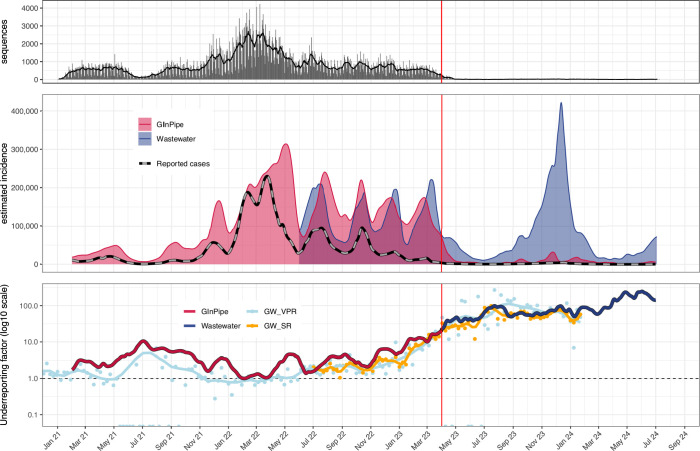
Integration of wastewater virus loads with GInPipe’s incidence estimates and validation for Germany. The upper panel shows the number of viral sequences per day in Germany (black bars = raw data, black line = 7-days rolling average). The middle panel depicts the estimation of the GInPipe-based minimal number of SARS-CoV-2 infected individuals per day in Germany (red area; description in the Methods) and viral load measurements from wastewater, linearly scaled to the GInPipe-based incidence (blue area). The daily reported cases are given by the black and grey dashed line. The lower panel shows the estimated under-reporting factor (ratio of incidence estimate vs. reported cases) on a log10 scale. The red line shows under-reporting estimates for GInPipe for the time with sufficient sequencing data and continuing in blue with the scaled wastewater incidence estimates. The light blue and orange points, together with the respective smoothed lines, show the under-reporting factor estimated with GrippeWeb (citizen-science) data using the virologic positivity rate approach (GW VPR) and the self-reported testing approach (GW SR)^74^. The vertical red line indicates the chosen cut date at 01. April 23, when the available sequence data declined drastically from (> 10,000 to ≤ 100 sequences/week).

**Extended Data Fig. 4:**
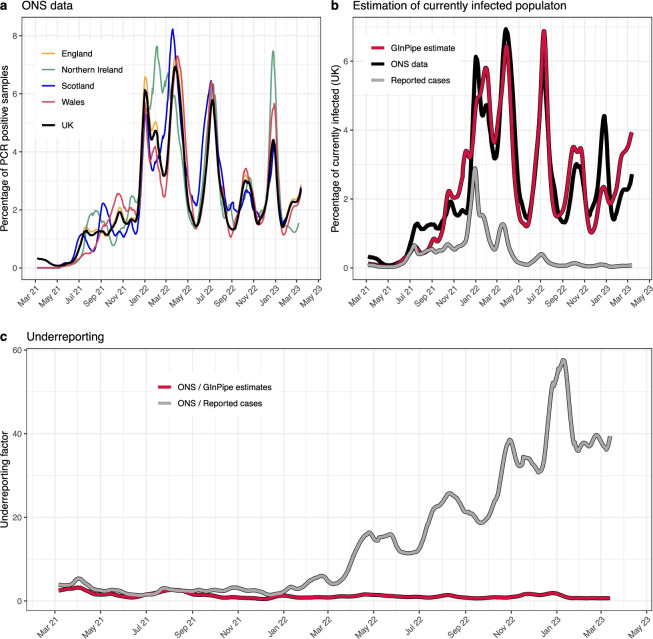
Validation of genome-based incidence estimates, using data from the UK. **a**. Average percentage of the population testing positive from the COVID-19 Infection Survey of the Office for National Statistics (ONS; ground truth data). Coloured lines depict the percentage for each UK constituent country. The black dashed line shows the average PCR-positivity (over all constituent countries) for the UK. **b**. Estimation of the SARS-CoV-2 positive population in the UK using ONS data (black line; ground truth), GInPipe’s estimate (red) scaled to the population (see Methods) and reported cases (grey), scaled to the population. A right-sided rolling sum of 10 days is applied to the reporting data and GInPipe’s estimates to approximate PCR-positivity. **c**. Under-reporting factor calculated as the ratio of the ONS data and the SARS-CoV-2 positive population fraction using reporting data (grey), and GInPipe’s incidence estimate (red). In line with previous work^75^, we performed separate GInPipe-incidence reconstructions for pre-omicron vs. omicron sequences (because of different evolutionary signals^76–79^, which are utilized in GInPipe^22^). The pre-omicron vs. omicron estimates were then scaled and added together to compute total incidence.

To overcome increasingly unreliable case reporting data, we reconstructed SARS-CoV-2 incidence trajectories from time-stamped viral genomes using the recently developed computational tool GInPipe^22^ in combination with available wastewater viral load data. In principle, any unbiased incidence estimate could be used. Here we used GInPipe for the time horizon 1 July 2021 to 31 March 2023, for which substantial amounts of viral genomic sequencing data are available (≈10,000 sequences per week). We then use wastewater viral load data for the remainder of the period, during which sequencing efforts dropped massively (<100 sequences per week from April 2023; Extended Data Fig. 3). We used the temporal overlap between wastewater virus load data and GInPipe incidence reconstructions (1 June 2022 to 31 March 2023) to estimate incidences from wastewater virus loads and to cross-validate incidence predictions (Extended Data Fig. 3). As an additional validation of the incidence reconstruction method, we show in Extended Data Fig. 4 that official case numbers substantially under-report the actual infection trajectory from the COVID-19 Infection Survey of the Office for National Statistics^6^ in the UK and show that GInPipe estimates are coherent with Office for National Statistics data and overcome under-reporting biases.

**Fig. 3:**
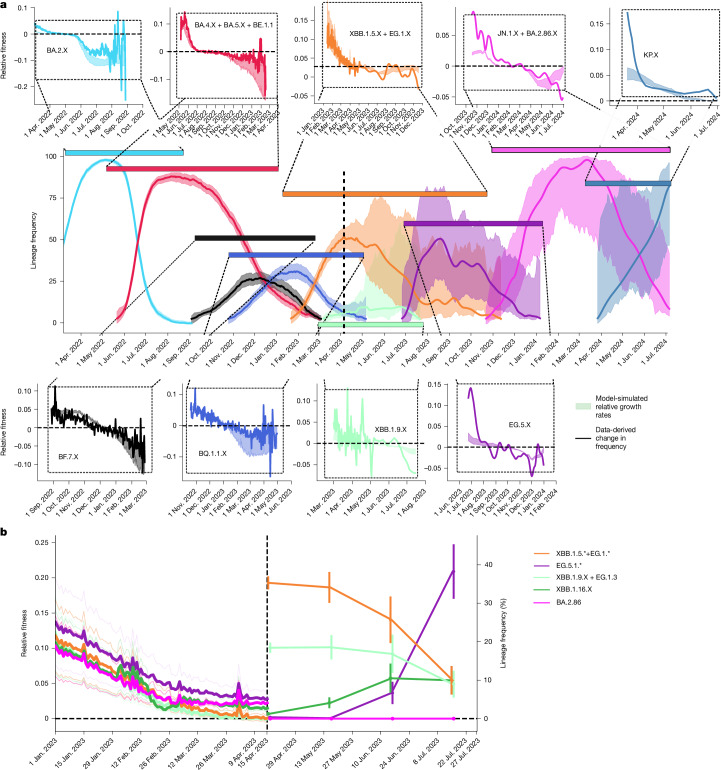
Dynamic immune landscape and variant dynamics in Germany. **a**, Historical variant dynamics in Germany during 1 March 2022 to 1 July 2024 of spike pseudo-groups BA.2,.X BA.5.X (+ BA.4.X + BE.1.1), BF.7.X, BQ.1.1.X, XBB.1.5.X (+ EG.1.X), XBB.1.9.X, EG.5.X, JN.1.X (+ BA.2.86.X) and KP.X, where ‘.X’ denotes inclusion of the respective sublineages. Confidence intervals were computed using Wilson’s method. Insets show model-predicted relative fitness *γ*_*y*_(*t*) (representing the relative number of susceptible individuals; coloured areas), with superimposed changes in frequency (*π*_*y*_(*t* − 1)/*π*_*y*_(*t*) − 1) seen in the data (solid lines). Note that the sequencing intensity changed markedly in April 2023 (vertical dashed line) as highlighted in Extended Data Fig. 3. **b**, Predicted variant dynamics. Left: model-predicted relative growth advantage *γ*_*y*_ of emerging spike pseudo-groups XBB.1.5* (+ EG.1*), XBB.1.9.X (+ EG.1.3), XBB.1.16.X, EG.5.1* and BA.2.86 (colours in key) using data until 15 April 2023 (asterisks (for example, EG.1*) indicate spike pseudo groups; that is, the group of lineages with identical mutations in the spike protein). Note that BA.2.86 was first detected on 24 July 2023 in Denmark and represents the dominating lineage (together with daughter lineage JN.1 globally, as of January 2024). Thick lines show average values, and light lines show minimum–maximum ranges resulting from pharmacokinetic variability. Right: data-derived lineage frequencies in the time after the prediction horizon (15 April 2023 to 27 July 2023). Confidence intervals (95%) were calculated using Wilson’s method with sample size *n* = 2,919, 500, 165 and 164 for April, May, June and July, respectively. Source Data

Next we reconstructed the timeline of all lineage frequencies, as illustrated for the most abundant groups in Fig. 3a. If SARS-CoV-2 evolution is driven by population immunity, the relative fitness of a variant will be determined by the expected number of individuals who are susceptible to the variant, relative to the expected number of susceptible individuals across competing (that is, simultaneously circulating) variants. In other words, on the basis of immunity, the relative fitness will be determined by whether the variant can infect more (or fewer) individuals than the current viral population. To test this hypothesis, we computed the expected number of susceptible individuals for each variant by integrating infection history, cross-neutralization and immune waning (Fig. 1 and Methods). We calculated this immunity-driven relative fitness from our model and compared the predictions with historical changes in variant frequencies (Fig. 3a). We found a strong match between our predictions and the real-world data: our model predicts the inflection point for BA.2 in April–May 2022, for the BA.4 + BA.5 pseudo-group between July and October 2022, and for the variants BF.7, BQ.1.1, XBB.1.5, XBB.1.9 and EG.5 in December 2022, January, mid-April, summer and autumn 2023, respectively (compare areas (predictions) versus lines (data) in the insets of Fig. 3a). For the more recent variants JN.1 and KP.1, KP.2 and KP.3, the model correctly predicts the inflection points during spring and summer 2024, respectively. Moreover, the data-derived change in variant frequency and our model-predicted immunity-driven relative fitness correspond in magnitude (see Methods for a theoretical justification). Notably, availability of sequencing data in Germany changed markedly in April 2023 (vertical dashed line in Fig. 3a,b and highlighted in Extended Data Fig. 3), which strongly affected confidence intervals regarding lineage frequency.

We next evaluated whether our model could forecast variant dynamics by using a dataset that ends on 16 April 2023. On the basis of this dataset, we evaluated the immunity-mediated relative growth advantage of the circulating variants XBB.1.5 (+ EG.1), XBB.1.9 (+ EG.1.3), XBB.1.16, emerging variant EG.5.1 and variant BA.2.86 (‘pirola’), which was detected for the first time on 24 July 2023 (3 months after the dataset ended). We visualized these predictions together with the actual variant dynamics observed after 16 April 2023 in Germany in Fig. 3b. Our predictions indicate that XBB.1.5 and XBB.1.9 had no growth disadvantage by mid-April 2023, XBB.1.16 had a slight growth advantage and EG.5.1 had a substantial growth advantage. These forecasts precisely match the actual variant growth dynamics in Germany during April–July 2023: XBB.1.5 and XBB.1.9 had reached their inflection points and declined subsequently from 35% and 20%, respectively, to 5%, XBB.1.16 slightly increased from 2% to 10%, and EG.5.1 substantially increased from <1% to >30%. The not-yet-emerged variant BA.2.86 had a model-predicted growth advantage that is slightly lower than EG.5.1, but with an increasing trend in contrast to all other lineages. Notably, BA.2.86 and its daughter lineage JN.1 dominated in Germany and globally by January 2024.

## Global immune and variant dynamics

**Fig. 4:**
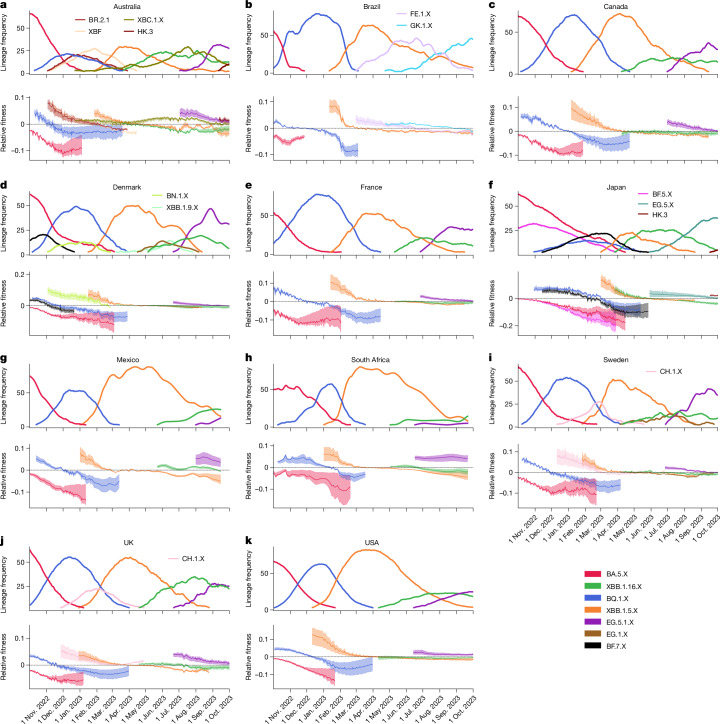
Dynamic immune landscape and variant dynamics across the globe. **a**–**k**, The relative abundance of major lineages when they surpass 3% relative abundance (solid lines, top) along with their model-calculated relative fitness *γ*_*y*_(*t*) (bottom lines represent mean estimates, shaded areas represent minimum–maximum intervals resulting from pharmacokinetic variability) for Australia (**a**), Brazil (**b**), Canada (**c**), Denmark (**d**), France (**e**), Japan (**f**), Mexico (**g**), South Africa (**h**), Sweden (**i**), the UK (**j**) and the USA (**k**). Note that in Brazil, the lineages BQ.1.X include BE.9, and lineages XBB.1.5.X include XBB.1.18.1. Source Data

**Extended Data Fig. 5:**
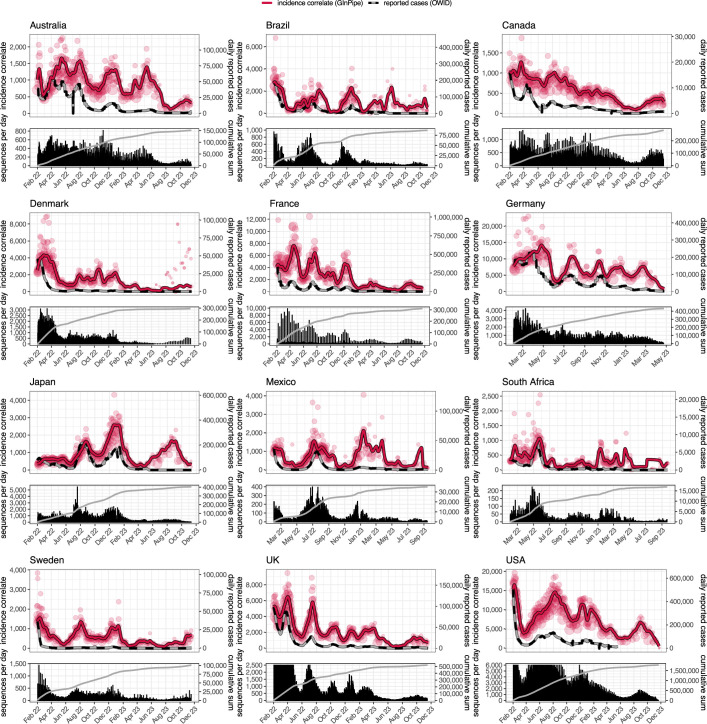
Incidence reconstruction for all considered countries. For each country, the upper panel shows the output of GInPipe (i.e., incidence correlates *ϕ*). The dots show the point estimates for each temporal sequence bin, while the red line depicts the smoothed incidence correlate *ϕ* (bandwith = 14). The black-grey line shows the reported (smoothed) SARS-CoV-2 cases for the respective country, acquired from OWID^80^. The lower panel shows the number of sequences per day in black bars, together with the cumulative number of sequences as a grey line.

**Extended Data Fig. 6:**
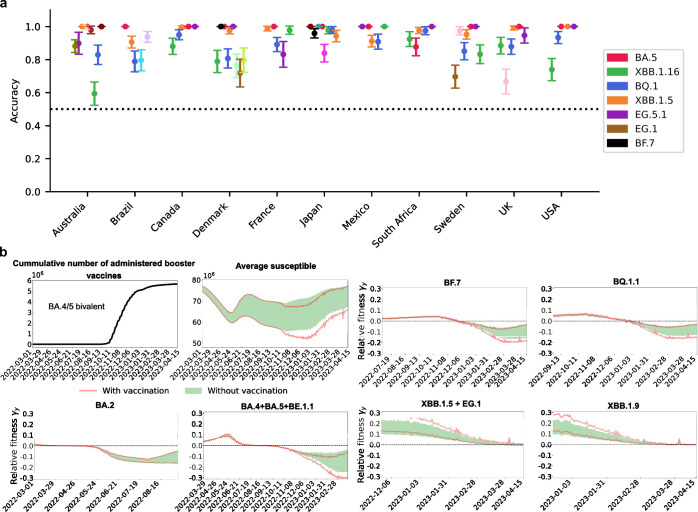
Accuracy of predictions per country and lineage (related to Fig. 4) and impact of BA.4/5 boosters. **a**. Each dot represents the accuracy of a specific lineage and country combination. Accuracy is determined by partitioning the frequency curve π*y* into days of rising (1) and falling (−1) trends, then comparing these with corresponding predictions γ_y_: If the full envelope is positive, the prediction is rising (1) if the full envelope is negative, the prediction is falling (−1): Days with negligible frequency changes or undecided predictions (envelopes with both positive and negative values) are excluded from the analysis. **b**. Predicted impact of bivalent (BA4/5) booster-vaccinations on the relative number of susceptibles (relative variant fitness) γ_y_ in Germany. Top left: Timeline of bivalent booster vaccine uptake in Germany (https://impfdashboard.de/) and average number of susceptibles (averaged with regards to the circulating ‘Spike pseudo-groups’ at the time). Remaining panels: Predicted relative fitness γ_y_ for major circulating variants in Germany March 2022–April 2023, without considering vaccinations (green area) and when considering both the timeline of infection and vaccinations respectively (red outlines).

We next evaluated our model to predict the adaptive immune landscape for 11 additional countries (Australia, Brazil, Canada, Denmark, France, Japan, Mexico, South Africa, Sweden, the UK and the USA). Essentially, we changed only the input of our model to the respective local infection history. In terms of data, this required only local genomic surveillance data, which we retrieved from GISAID (the Global Initiative on Sharing All Influenza Data; summary of data in Supplementary Table 6). We then used GInPipe^22^ to reconstruct the incidence timeline as shown in Extended Data Fig. 5, highlighting that case reporting changed over time for all countries and almost ceased at the beginning of 2023. The genomic surveillance data were then used to reconstruct lineage frequencies. From these two components (number of infections and lineage frequencies), we computed the time-dependent regional immune landscapes, which determine relative fitness of each lineage of interest. In Fig. 4, we depict lineage frequencies in the 11 countries together with model-predicted relative fitness *γ*_*y*_ for the 20 most common lineages during October 2022–October 2023. By utilizing the individual infection history in each country, the model predicts well whether a lineage is going to rise (relative fitness *γ*_*y*_ > 0) or decline (*γ*_*y*_ < 0) with an average accuracy of 0.92 (Extended Data Fig. 6a). Besides the three major lineages (BA.5, BQ.1 and XBB.1.5) that spread to some extent in all countries, our model was also able to predict the dynamics of some country-specific lineages, such as BR.2.1, XBC.1.3 and XBC.1.6 in Australia, FE.1 and GK.1 in Brazil, BN.1.X in Denmark, and HK.3 in Australia and Japan at the end of the investigated time horizon. Moreover, differences in the magnitude and duration of lineage-specific waves in the distinct countries became apparent, and the diversity of lineages increased towards the end of the investigated time horizon.

## Infection history dictates lineage success

**Fig. 5:**
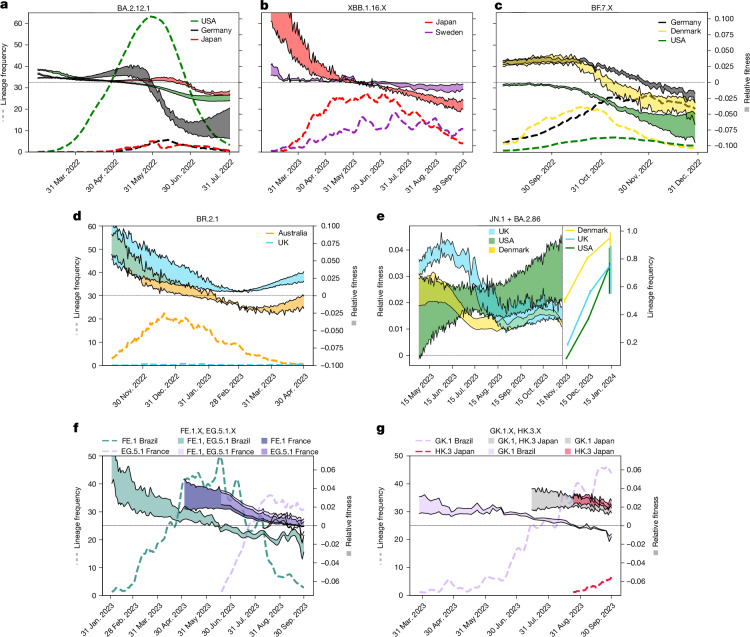
Country-specific immune landscape and effects on variant success. **a**, Dynamics of BA.2.12.1 in the USA (green dashed line) versus Germany (black dashed line) and Japan (red dashed line) along with the model-predicted relative number of BA.2.12.1 susceptible individuals (representing relative fitness *γ*_*y*_) in the USA, Germany and Japan (green, grey and red areas, respectively). **b**, Dynamics of XBB.1.16 in Japan and Sweden (red and magenta dashed lines, respectively) along with the model-predicted relative fitness (red and magenta areas, respectively). **c**, Dynamics of BF.7 in Germany, Denmark and USA (dashed black, yellow and green lines, respectively) along with the model-predicted relative fitness (grey, yellow and green areas, respectively). **d**, Dynamics of endemic variant BR.2.1 in Australia (orange dashed line) versus the UK (cyan dashed line) along with the corresponding model-predicted relative fitness (orange and cyan areas, respectively). **e**, Model-predicted relative fitness of BA.2.86 and JN.1 in the USA, UK and Denmark (green, cyan and yellow areas, respectively) up to mid-November 2023 and variant dynamics in the following 2 months (green, cyan and yellow, respectively; error bars represent mean frequency and 95% confidence interval computed using Wilson’s method). **f**, Dynamics of convergently evolved lineages FE.1 in Brazil (green dashed line) versus EG.5.1 in France (purple dashed line) and predicted relative fitness (shaded areas). **g**, Dynamics of convergently evolved lineages GK.1 (Brazil, purple dashed line) versus HK.3 (Japan, red dashed line) along with the model-predicted relative fitness (shaded areas). Source Data

Next we wanted to investigate why particular lineages were successful in only some, but not all countries. For example, BA.2.12.1 dominated in the USA, with variant proportions >50% between the beginning of May 2022 and mid-June 2022, but did not reach similar dominance elsewhere. Notably, BA.2.12.1 is a daughter lineage of BA.2 that acquired the alteration 452Q. To test our hypothesis that infection history and variant-specific immunity may have determined the success of BA.2.12.1 in the USA, but not elsewhere, we used our model to calculate its immunity-driven relative fitness (Fig. 5a). We observed that although BA.2.12.1 emerged in the USA as soon as March 2022, by the time it reached Germany and Japan (May 2022), its relative fitness was already declining and reached *γ*_*y*_ < 0 soon after, which predicts that the variant would be less fit than the average viral population, and fail to invade Germany and Japan. In other words, BA.2.12.1 could not spread in Germany and Japan, because it entered the country ‘too late’ and the preceding BA.2 wave (March to June or July 2022) had created substantial cross-neutralizing immunity.

We looked at XBB.1.16, an XBB.1.5 descendent with the 478K alteration, in Japan versus Sweden, as another example. Whereas the lineage quickly spread in Japan towards frequencies of 25% within 2 months, it rose only modestly to ≤15% in Sweden. Our model precisely predicted these dynamics, indicating that XBB.1.16 had a substantial immunological advantage in Japan that dissipated by the end of April 2023. By contrast, the lineage had only a modest model-predicted fitness advantage in Sweden, which vanished around the same time (Fig. 5b). An explanation of this model prediction (and observed variant dynamics) lies in the length of the preceding XBB.1.5 wave between the two countries: in Sweden, the XBB.1.5 (+EG.1) wave was substantial, creating cross-immunity towards XBB.1.16. By contrast, the corresponding infection wave in Japan contained a mix of lineages (compare also Fig. 4f,i and Extended Data Fig. 5), which left an immunological niche to be filled by XBB.1.16 in Japan.

A notable phenomenon can be observed with strain BF.7, which spread substantially in Germany and Denmark, but not in the USA (Fig. 5c). BF.7 is a descendent of BA.5 that acquired an alteration at 346T (an alteration that occurred in several independent lineages by convergent evolution^23,24^). On the first appearance of BF.7, its predicted relative fitness is low in the USA, and in fact, BF.7 occurs less frequently in the USA than in Germany or Denmark. At the same time, BQ.1 is circulating in the USA, but not in Germany or Denmark. BQ.1 seems to better fill the existing immunological niche in the USA. Although BQ.1 never succeeds in these two European countries, BQ.1.1, which denotes a BQ.1 daughter lineage that acquired 346T, occurs instead, putting an end to the further spread of BF.7 (compare insets in Fig. 3a).

In Australia, lineage BR.2.1 replaced BQ.1.X from November 2022 as predicted by our model (Figs. 4a and 5d). Both lineages evolved alterations at the same positions within the RBD (339 and 486), but the respective alterations are distinct (339H and 486I in BR.2.1, and 339D and 486V in BQ.1.1). Moreover, whereas BR2.1 acquired 446S, BQ1.1 acquired an alteration at 444T. However, the main difference is that BR.2.1 emerged from BA.2.75 with five alterations in the antigenic super-site of its NTD that are not present in BA.5 descendent strains, such as BQ.1.X. Considering that a massive wave of BA.5 preceded the emergence of BR.2.1, it is not unexpected to find (and predict) that BR.2.1 would initially have a larger immunological niche than BQ.1.X, as predicted for Australia. This raises the question of whether BR.2.1 would have spread elsewhere. Our model predicted that it could have (for example, in the UK). However, only sporadic BR.2.1 imports occurred, with all infection chains ending, before the variant could overtake (Fig. 5d).

Next, we wanted to assess whether our model would predict the dynamics of the emerging lineage BA.2.86 (and JN.1) at the end of 2023. This lineage lacks many alterations that were previously acquired in convergently evolved lineages (for example, 346T, 368I, 445P, 455F, 456L and 490S), but instead harbours a set of unique alterations in the NTD and RBD. When taking the end of our investigated timeline into account (mid-November 2023), our model predicts that BA.2.86 (+JN.1) would increase in frequency in all investigated countries (illustrated in Fig. 5e for a selection of countries). As predicted by our model, BA.2.86 and JN.1 were steeply on the rise from mid-November 2023 until January 2024. Our model even predicted the magnitude of increase, for which the steepest increase is observed in the USA, followed by the UK and Denmark.

Last, we looked at convergent evolution in different regions of the globe. For example, FE.1 and EG.5.1 dominated in Brazil versus all other investigated countries. They share an almost identical RBD and NTD alteration profile, but emerged from XBB.1.18.1 versus XBB.1.9, respectively. Our model estimates nearly identical relative fitness for these two lineages in the individual countries. FE.1 rose to dominance in Brazil in May 2023 and started to be replaced by July 2023, as predicted by our model. FE.1 was never really exported in considerable quantities to Europe, the USA or South Africa, leaving the immunological niche to be filled by EG.5.1, which appeared from August onwards and rose to dominance. However, EG.5.1 could never successfully invade Brazil, as FE.1 had already created cross-immunity. Likewise, GK.3 (an XBB.1.5 descendent) rose in Brazil after the FE.1 wave. As predicted by our model, HK.3 (an EG.5 descendent) with an identical NTD and RBD profile compared to GK.3 rose in Japan and Australia after the respective EG.5.1 wave.

These numerous applications of our model highlight that we can identify whether an emerging variant may encounter an immunological niche in a particular country that favours its growth. Thus, the model may be suitable to serve as a variant alert system that can be applied to the regional infection history.

## Discussion

Exposure to SARS-CoV-2 triggers adaptive immune responses, which, in simplified terms, comprise a cellular immune response (CD4^+^ and CD8^+^ T cell responses) and a humoral immune response (B cell-associated antibody production)^25,26^. Although T cell immunity may contribute to an overall decline in SARS-CoV-2 transmissibility and severity^27,28^, it may not differentiate between variants, as variants share T cell epitopes^29^, and T cells are often cross-reactive^30^. Among the produced antibodies, only those targeting epitopes in the RBD and NTD of the spike protein, the main site of viral evolution, are considered virus neutralizing^31^. Herein, we developed a mechanistic model, combining regional infection history and variant cross-neutralization based on RBD and NTD alteration profiles, to predict an adaptive immune landscape. This landscape describes the relative number of susceptible individuals for any SARS-CoV-2 variant, determines its relative fitness and dictates variant dynamics (Figs. 3–5).

In our study, we utilized DMS data to compute cross-neutralization between any pair of variants, including those that have not yet appeared (Figs. 3b and 5e). Combining these data with incidence reconstruction from virus sequencing data^22^, or a combination of unbiased incidence measures (such as viral load measurements in wastewater^32^; Extended Data Figs. 3 and 4), we could overcome limitations of earlier works^3^. Overall, the developed method can accurately model regional immune landscapes that were shaped by the infection history of all (≫100) locally encountered variants, explaining country-specific differences in evolutionary dynamics (Fig. 5) and including countries with regionally endemic variants (for example, BR.2.1 in Australia, and FE.1 and GK.1 in Brazil; Fig. 5d,f,g).

## Limitations

Although the developed model has minimal data requirements, genomic surveillance data need to be available to contextualize the risk of occurrence and spread of variants to a regional scale. On the basis of a sensitivity analysis for the German dataset (Extended Data Fig. 8), we recommend at least 100 randomly sampled sequences per week.

Our model relied on a number of assumptions and simplifications. We did not include seasonality, because it is irrelevant for determining the relative variant-specific fitness (it cancels out). Although there is still no scientific consensus regarding the seasonality of SARS-CoV-2 incidence^33^, a recent study of influenza and endemic coronaviruses suggested that the availability of susceptible individuals may be a dominant driver for infection incidence^34^.

**Extended Data Fig. 7:**
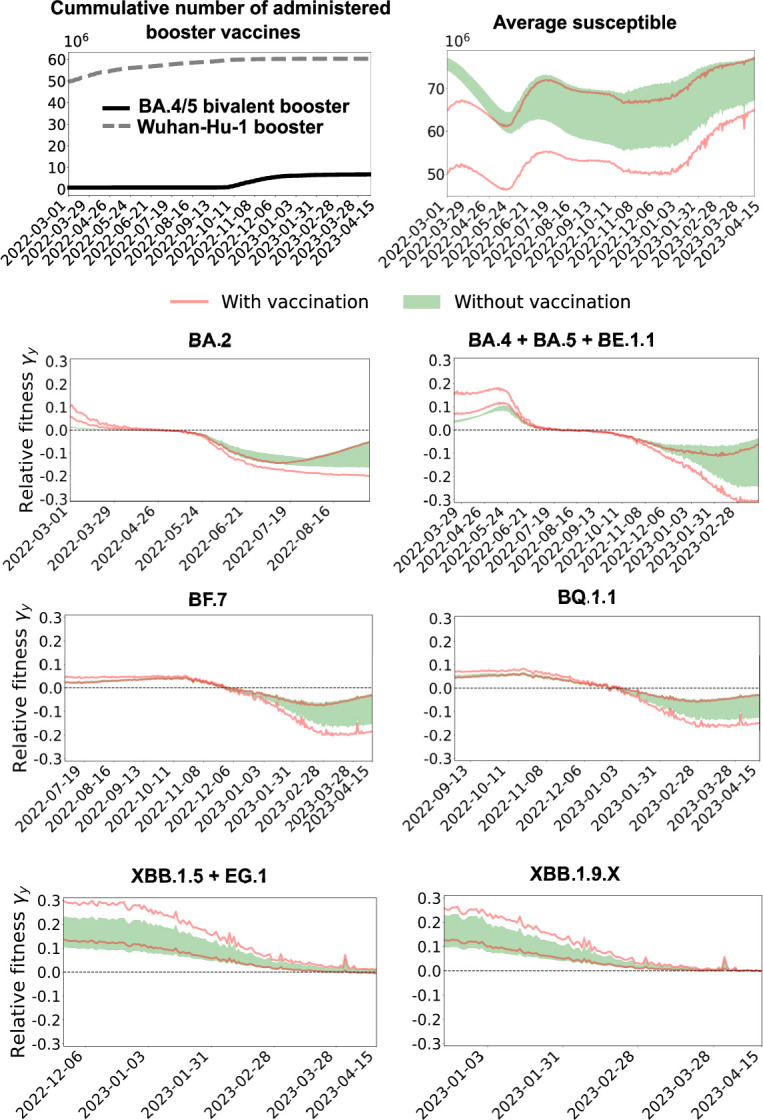
Predicted impact of ≥3rd booster-vaccinations on the relative number of susceptibles (relative variant fitness) γ_y_ in Germany. Top left: Timeline of booster vaccine uptake in Germany (https://impfdashboard.de/). Top right: Average number of susceptibles (averaged with regards to the circulating ‘Spike pseudo-groups’ at the time). Predicted relative fitness γ_y_ for major circulating variants in Germany March 2022–April 2023, without considering vaccinations (green area) and when considering both the timeline of infection and vaccinations respectively (red outlines).

**Extended Data Fig. 8:**
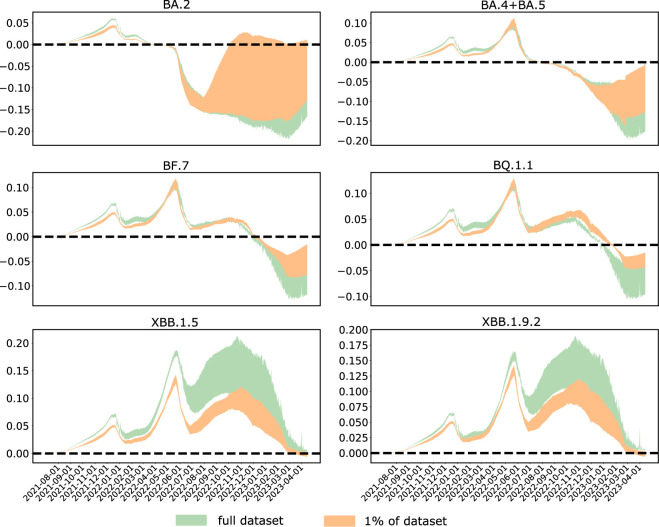
Sensitivity of the developed method to diminishing data input. We down-sampled the sequence data to 1% for Germany and superimposed model predictions for the relative variant fitness γ_y_ using the full dataset (green areas) vs. 1% of the data (orange areas).

We did not consider the timeline of vaccination, as we found no impact on relative fitness estimates *γ*_*y*_(*t*) (Extended Data Fig. 6b) for the considered time horizon. This observation can be explained by the limited uptake of BA.4 + BA.5-adapted booster vaccines (for example, ≈5.6 million doses in comparison to ≈23.4 million BA.4 and BA.5 infections in Germany). For the ≈60 million Wuhan-Hu-1-based third-dose boosters in Germany, we observed that they had a scaling effect on *γ*_*y*_(*t*) that vanished by 2023 (Extended Data Fig. 7). Wuhan-Hu-1-based booster vaccinations removed susceptible individuals similarly across all Omicron variants, inducing stronger competition (higher amplitude of *γ*_*y*_(*t*)), without affecting the order of *γ*_*y*_(*t*) estimates, or inflection point predictions. As vaccine uptake further declined from 2023 and infection dynamics remained at a high level (Extended Data Fig. 5), our observation strongly suggested that it is not necessary to consider vaccinations to estimate relative fitness from 2023 onwards (Figs. 4 and 5). However, it will probably be crucial to consider vaccination uptake when evaluating case severity and hospitalization risks, which is beyond the scope of the developed model; the model does not consider cellular immune responses, which are an important determinant of clinical severity.

Notably, as the scope of our model is the prediction of the (humoral) immune pressure at a population level, we consider the average immune dynamics, which may not predict individual protection profiles that emerged from a complex sequence of antigen exposures^35^. Likewise, our model is not suitable for estimating an absolute protective antibody titre against SARS-CoV-2 infection as it operates with unitless antibody concentrations.

In predicting variant phenotypes, we assumed that antibody-evasive mutational effects are mostly additive^36^, which allowed us to utilize DMS data to quantify the effect of antibody-evasive alterations in complex evolutionary backgrounds (compare Extended Data Fig. 1d,e–g). This assumption is coherent with cases in which compensatory alterations enable the emergence of antibody-evasive alterations and in which there is no direct epistatic interaction between antibody-evasive alterations^37^. However, machine-learning-based approaches could in the future be utilized^38^ instead of DMS, to capture more complex epistatic interactions. Likewise, protein language models^39^ may be used to forecast the acquisition of additional alterations in emerging variants and, when combined with our work, enable us to extend its prediction horizon (compare Fig. 3b).

## Conclusion

Overall, our work provides proof that the ongoing evolution of SARS-CoV-2 is driven by variant-specific population immunity. Moreover, the developed model constitutes a means to calculate the dynamic immune landscape that resulted from recent infection history and determines variant fitness.

In the future, our approach could serve as a basis to identify epitopes most likely to be under recent selective pressure and, therefore, provide cues for designing vaccine candidates that maximize neutralization breadth against emerging variants in a forthcoming season. For this, previously developed approaches in the context of influenza H3N2 vaccine design^40,41^ could be complemented using the SARS-CoV-2 fitness models developed herein. Furthermore, our conceptual ideas may be transferred to model the evolution of other respiratory viruses that are subject to molecular surveillance.

## Methods

### DMS data

We used all DMS data^5,17^ available on 13 February 2023 to assess the phenotype (escape from neutralizing antibodies) of each SARS-CoV-2 variant. DMS measures, in a yeast-display assay, how much a mutated site *s* in the RBD affects the binding of antibodies elicited by a variant that is not mutated at site *s*. We utilized data from 836 antibodies that were classified into 12 distinct epitope classes^17,18^ (see below) and aggregated all values on site level by their mean, yielding ‘escape fractions’ to antibody *a* for each mutated site ef_*s*,*a*_ (values were bounded at 0.99). Escape fractions denote a proxy for quantifying the probability that an antibody does not bind an RBD that contains an alteration at site *s*. Thus, the numerical value depends on the antibody potency, and hence we aimed to convert these values to ‘fold resistances’ (fold change in antibody potency; see also Extended Data Fig. 1a–c). Assuming that mutational effects of site *s* on the binding affinity are independent, the binding probability of an antibody elicited by a variant *x* to a variant *y* can be expressed as

1 $${b}_{a}(x,y)=\,\prod _{s\in \varOmega (x,y)}\,(1-{{\rm{ef}}}_{s,a})\,,$$

in which ef_*s*,*a*_ is the normalized escape fraction of mutated site *s* with respect to antibody *a*, and $\varOmega (x,y)$ denotes the set of RBD sites that distinguish variants *x* and *y* (ref. ^17^). Using classical pharmacodynamic approaches, we then model the binding probability as

2 $${b}_{a}(x,y)=\frac{{c}_{a}}{{{\rm{FR}}}_{x,y}(a)\,\cdot {{\rm{IC}}}_{50({\rm{DMS}})}(a)+{c}_{a}},$$

in which FR_*x,y*_(*a*) denotes the fold resistance of variant *y* to an antibody *a* elicited by variant *x*. The parameter ${{\rm{IC}}}_{50({\rm{DMS}})}(a)$ corresponds to the half-maximal inhibitory concentration (that is, ‘potency’) of the antibody against the antibody-eliciting variant, which was extracted from the DMS dataset. Notably, *c*_*a*_ = 400 μg ml^−1^ was the antibody concentration at which the DMS experiment was conducted^4,18^. Combining equations (1) and (2) yields:

$${{\rm{FR}}}_{x,y}(a)=\frac{{c}_{a}}{{{\rm{IC}}}_{50({\rm{DMS}})}(a)}\left(\frac{1}{{b}_{a}(x,y)}-1\right).$$

As already evident from Extended Data Fig. 1a–c, DMS estimates of ef_*s*,*a*_, as well as the corresponding FR_*x*,*y*_(*a*), can become unreliable depending on antibody concentrations and antibody potency ${{\rm{IC}}}_{50(x)}(a)$, falsely predicting hyper-susceptibility (FR_*x,y*_(*a*) < 1). We enforce that FR_*x*,*y*_(*a*) ≥1 to avoid such artefacts.

### Epitope classes

On the basis of the similarity of antibody profiles in DMS data, antibodies were previously classified into 12 epitope classes (A, B, C, D1, D2, E1, E2.1, E2.2, E3, F1, F2, F3)^18^ (Supplementary Tables 1 and 2). As we encountered more than 30% missing values for epitope classes E2.1 and E2.2, we merged them with E1 into a new class (E12), as they bind to similar regions, including amino acid site R346 (ref. ^42^). Finally, we retrieved a matrix of 10 epitope classes *Α* = (A, B, C, D1, D2, E12, E3, F1, F2, F3) for 179 RBD sites. This classification indicates that antibodies belonging to the same class bind to overlapping epitopes, and there is little overlap between epitope classes (Fig. 2a). Consequently, we assumed that antibodies within the same epitope class would be similarly affected by RBD alterations, whereas phenotypic changes between epitope classes may vary considerably.

We then quantified the fold resistance associated with each epitope class as the average fold resistance of all antibodies belonging to the class

$${{\rm{F}}{\rm{R}}}_{x,y}({\vartheta })={\rm{m}}{\rm{e}}{\rm{a}}{\rm{n}}({{\rm{F}}{\rm{R}}}_{{\rm{x}},{\rm{y}}}({\rm{a}}):{\rm{a}}\in {\vartheta }),$$

for all epitope classes ${\vartheta }$ in *Α* (Fig. 2a).

As a proof of concept, we compared DMS-derived FR_*x*,*y*_(${\vartheta }$) using the calculations above with fold resistance values obtained from virus neutralization assays (reported in ref. ^42^) for antibodies targeting all epitope classes *Α* defined above. As can be seen in Extended Data Fig. 1d, we observed a strong and significant positive correlation between the DMS-derived FR_*x,y*_(${\vartheta }$) and those obtained by neutralization assays and reasonable agreement for polyclonal sera (Extended Data Fig. 1e–g).

As the DMS experiments were generated only for RBD-targeting antibodies, no escape data were available to quantify the fold resistance of NTD-targeting antibodies. To overcome this limitation, we included an additional class of NTD-targeting antibodies targeting three antigenic super-sites^43^: spike amino acid positions 14–20, 140–158 and 245–264. Consequently, we assigned alterations in the antigenic super-sites fold resistance values of 10, which is in range with corresponding ELISA experiments^43^. However, the model can be updated if comprehensive DMS data for the NTD domain become available^44^.

Assuming independence between mutational effects, the total fold resistance of a variant *y* to binding of an NTD-targeting antibody elicited by a variant *x* was computed as:

$${{\rm{FR}}}_{x,y}({\rm{NTD}})={10}^{| \varOmega (x,y)| }\,,$$

in which *|Ω*(*x*, *y*)*|* denotes the number of mutational differences between variants *x* and *y* in the antigenic super-site of the NTD.

### Variant cross-neutralization probability

We assumed that a virus is neutralized if at least one antibody is bound to its surface (either at the RBD or NTD of the spike protein). Here, we collectively consider all antibodies from the same epitope class as they compete for the same binding site. By assuming binding independence between epitope classes, the neutralization probability can be computed as:

$${P}_{{\rm{N}}{\rm{e}}{\rm{u}}{\rm{t}}}(t,x,y)=1-\prod _{{\vartheta }\in {A}_{x{\rm{\setminus }}y}}(1-{b}_{{\vartheta }}(t,x,y)),$$

with ${b}_{{\vartheta }}(t,x,y)$ denoting the probability that an antibody of epitope class ${\vartheta }$ in *Α* ∪ (NTD) binds to the virus with

$${b}_{{\vartheta }}(t,x,y)=\frac{{c}_{{\vartheta }}(t)}{{{\rm{FR}}}_{x,y}({\vartheta })\cdot {{\rm{IC}}}_{50(x)}({\vartheta })+{c}_{{\vartheta }}(t)},$$

in which ${c}_{{\vartheta }}(t)$ is the antibody’s concentration in an individual at time *t*, ${{\rm{IC}}}_{50(x)}({\vartheta })$ is the half-maximal inhibitory antibody concentration against the variant that elicited the antibody. FR_*x*,*y*_(${\vartheta }$) is the fold resistance of variant *y* to binding of antibodies of epitope class ${\vartheta }$, elicited by variant *x*.

### Antibody potency

Next, we quantified ${{\rm{IC}}}_{50(x)}({\vartheta })$ for each epitope class. As the DMS data were derived from yeast-display RBD mutant libraries, absolute antibody potencies may not directly translate to a clinical setting. However, the ranking of antibody potencies may be preserved. Consequently, we estimated the relative potency *D*(${\vartheta }$) from the DMS data:

$$D({\vartheta })=\frac{\widehat{\,{{\rm{IC}}}_{{50}_{({\rm{DMS}})}}}({\vartheta })}{\frac{1}{| {\rm{A}}| }{\sum }_{\zeta \in {\rm{A}}}\widehat{\,{{\rm{IC}}}_{{50}_{({\rm{DMS}})}}}(\zeta )},$$

in which ${\vartheta }\in {\rm{A}}$, and $\widehat{\,{{\rm{IC}}}_{{50}_{({\rm{DMS}})}}}({\vartheta })$ denotes the average potency of all antibodies belonging to epitope class ${\vartheta }$. Epitope-class-specific clinical antibody potency ${{\rm{IC}}}_{50(x)}({\vartheta })$ was then inferred using the following relation:

$${{\rm{IC}}}_{50(x)}({\vartheta })=D({\vartheta })\cdot \widehat{\,{{\rm{IC}}}_{{50}_{(x)}}},$$

in which $\widehat{\,{{\rm{IC}}}_{{50}_{(x)}}}$ denotes the ${{\rm{IC}}}_{50(x)}$ averaged over all epitope classes. NTD-targeting antibodies were not included in the DMS dataset, and hence we set

$${{\rm{IC}}}_{50(x)}({\rm{NTD}})=\,\widehat{\,{{\rm{IC}}}_{{50}_{(x)}}}.$$

$\widehat{\,{{\rm{IC}}}_{{50}_{(x)}}}$ was the only free parameter in the model, which we estimated by fitting our model to (Wuhan-Hu-1-strain) vaccine efficacy (VE) data against the Delta lineage (B.1.617.2) present between 4 July 2021 and 31 December 2021 (Fig. 2d; genomic profile in Supplementary Table 3).

We considered interindividual differences in antibody pharmacokinetics (see below), implemented as combinations of the parameters *t*_max_ (time of maximal antibody concentration) and *t*_half_ (antibody half-life). For parameter estimation, we first estimated optimal drug potencies $\widehat{\,{{\rm{IC}}}_{{50}_{(x)}}}$ (*t*_max_, *t*_half_) for each *t*_max_, *t*_half_ combination in a 5 × 15 grid (ranges below) and then averaged over these 75 estimates. Parameter estimation was performed using scipy.optimize.root, applying the Levenberg–Marquardt method to solve the ordinary least-square problem.

$$\mathop{{\rm{argmin}}}\limits_{\widehat{\,{{\rm{IC}}}_{{50}_{(x)}}}({t}_{\max },{t}_{{\rm{half}}})}\sum _{t}\parallel {{P}_{{\rm{Neut}}}(t,\text{Wuhan-Hu-1},\text{Delta and}\widehat{{{\rm{IC}}}_{{50}_{(x)}}}({t}_{\max },{t}_{{\rm{half}}}))-{\rm{VE}}(t,\text{Wuhan-Hu-1},{\rm{Delta}})\parallel }^{2},$$

in which VE(*t*, Wuhan-Hu-1, Delta) denotes the vaccine efficacy against the Delta strain *t* days after antigen exposure with the Wuhan-Hu-1 strain. Here, we assumed that VE = infection risk reduction ≈ *P*_Neut_.

As a proof of concept, we then tested our predictions with Wuhan-Hu-1-strain VE data against Omicron infection (Fig. 2d; genomic profile in Supplementary Table 3). Utilized VE data include those from all studies in which Wuhan-Hu-1-strain vaccines were tested and which were computed on the basis of hazard ratios or rate of confirmed infection (Supplementary Tables 4 and 5).

### Antibody pharmacokinetics

To determine the duration of sterilizing immunity against any variant *y*, we accounted for antibody pharmacokinetics (PK) after antigen exposure to variant *x* (by means of infection or vaccination). Pharmacokinetics were considered using a classical, descriptive linear model with an analytical solution

$$c(t)=\frac{{{\rm{e}}}^{-{k}_{e}t}-{{\rm{e}}}^{-{k}_{a}t}}{{{\rm{e}}}^{-{k}_{e}{t}_{\max }}-{{\rm{e}}}^{-{k}_{a}{t}_{\max }}},$$

in which *t* denotes the time after antigen exposure and *c*(*t*) denotes the normalized (fraction of maximum) concentrations of the antibody. The parameters *k*_*e*_ and *k*_*a*_ (elimination and ‘absorption’ parameters in classical PK models) were related to known quantities through established PK relations; that is,

$${k}_{e}=\frac{{\rm{ln}}(s)}{{t}_{{\rm{half}}}},\,{t}_{\max }=\frac{{\rm{ln}}\left(\frac{{k}_{a}}{{k}_{e}}\right)}{{k}_{a}-{k}_{e}}.$$

In our simulations, we considered identical PK for antibodies of the different epitope classes. Utilized parameters (*t*_max_, *t*_half_) were extracted from the literature: the time of maximal antibody concentrations (*t*_max_) varied between 14 and 28 days after antigen exposure^26,45,46^, and the half-life (*t*_half_) ranged between 25 and 69 days^19,47–53^. For simulation, we used different combinations of (*t*_max_, *t*_half_) in a 5 × 15 grid within a range of *t*_max_ within 14 to 28 days and *t*_half_ within 25 to 69 days and plotted the range of predictions (minimum, maximum).

### SARS-CoV-2 genomic data

We collected SARS-CoV-2 genomic data from Germany published by the Robert Koch Institute, including only genomic data from the ‘random sampling’ strategy, which denotes most of the sequence data. For other countries (Australia, Brazil, Canada, Denmark, France, Japan, Mexico, South Africa, Sweden, the UK and the USA), we downloaded data from GISAID (https://gisaid.org); however, here we could not be sure that the data are representative (randomly sampled), as anyone can upload SARS-CoV-2 data to GISAID. A dataset summary is given in Supplementary Table 6.

### Variant proportions and spike pseudo-groups

If pangolin lineage information was absent in the data, lineage information was assigned using established methods^54,55^. Alteration profiles for all sequences were extracted using covSonar. For each lineage, we collected all ‘characteristic’ spike amino acid changes in the RBD (amino acid position 331–541) and the NTD antigenic super-sites regions (amino acid positions 14–20, 141–158 and 245–264) for subsequent analyses. In our work, ‘characteristic’ implied that an amino acid change was present in at least 75% of all sequences from that lineage. The ‘antigenic profile’ for each lineage was then determined on the basis of the set of unique alterations within the NTD and RBD regions. Differences between lineages were defined as the set difference between alteration profiles. Clustering lineages with identical ‘antigenic profiles’ yielded spike pseudo-groups with distinct genomic profiles in the RBD and NTD region of the spike protein. On the basis of the genomic profiles and their clustering into spike pseudo-groups, we computed pseudo-group frequencies *π*_*x*_(*t*) for all *x* ∈ ${\mathcal{X}}$ in the entire observation horizon. The frequencies were computed such that there were at least 100 sequences per time step, and daily lineage frequencies were computed by using linear interpolation between time steps. Furthermore, we filtered out spike pseudo-groups that never reached levels of >1% frequency to reduce noise from sequencing errors. Data files with lineage and pseudo-group frequencies and alteration profiles for all countries are available via GitHub at https://github.com/KleistLab/VASIL (a summary is given in Supplementary Table 6).

### Genome-based reconstruction of infection timeline

Unfortunately, the infection timeline cannot be reconstructed from reported cases, as test coverage and reporting of SARS-CoV-2 became increasingly unreliable. To overcome this data limitation, we recently developed the genome-based incidence estimator, GInPipe^22^. This computational pipeline reconstructs the infection timeline solely from time-stamped viral sequences. Owing to the short infectious phase of SARS-CoV-2 (ref. ^56^), a limited amount of intra-patient evolution is typically observed before an infection is passed on. This implies the existence of an ‘evolutionary signal’ *ϕ* that relates haplotype diversity and the number of alterations present in a temporal collection of viral sequences to the number of infections (see ref. ^22^ for details). We confirmed previously that this evolutionary signal is proportional to the actual number of infected individuals *I*(*t*) *≈* *cϕ*(*t*) at time *t*.

Although GInPipe works quite robustly when sequencing efforts change over time, extreme changes may cause biases^22^. For the USA and the UK, we observed major drops in the number of available sequences (≈10-fold after 2022), and henceforth downsampled the number of viral sequences to 6,000 and 2,500 per day, respectively, which corresponds to the maximum number of sequences after the drop in sequencing efforts (Extended Data Fig. 5).

In the pipeline, sequences are pooled according to their collection date, such that ‘bins’ b comprise either the same number of sequences *n*_b_ or span the same number of days ∆*d*_b_. We chose time spans ∆*d*_b_ = 7, 10 and 14 days and bin sizes of *n*_b_ = 2% and 5% of all sequences, as well as the average weekly number of sequences for each particular country. Incidence correlates *ϕ*_b_ were filtered out if the time span of a bin was smaller than 3 days, or if a bin comprised fewer than 50 sequences. We allowed a bin to span at most 21 days (data-rich setting) or as many days as necessary to comprise the minimal number of sequences (data-poor settings). Bin-wise *ϕ*_b_ estimates were smoothed using kernel smoothing with a bandwidth of 14. GInPipe-estimated incidence correlates are depicted in Extended Data Fig. 5 for all investigated countries.

To confirm the validity of GInPipe-estimated incidences, we compared our predictions with other unbiased incidence measures for Germany (citizen science and wastewater data; Extended Data Fig. 3) and with a UK dataset from the representative COVID-19 Infection Survey of the Office for National Statistics (ONS)^6^, using the percentage of infected individuals in the UK from the ONS study as the ground truth (March 2021–March 2023; Extended Data Fig. 4).

The percentage of PCR-positive individuals from GInPipe at the time point of interest was then computed using a rolling sum over 10 days^56^, and normalized with the ratio of the population size and the sum of infected people in the respective time horizon according to the ONS. For the reported cases, the rolling sum over 10 days was normalized by the population size of the UK.

### Expected sterilizing immunity against variant *y*

The estimation of cross-neutralization probability *P*_Neut_ enabled us to estimate the expected number of individuals being immune against infection with a variant *y* for a given time point *t* by taking the infection history before time *t* into account. The expected number of individuals immune to infection with strain *y* is given by

$${\mathbb{E}}[{{\rm{Immune}}}_{y}(t)]=\,\sum _{x\in {\mathcal{X}}}\underset{0}{\overset{t}{\int }}{\pi }_{x}(s)\cdot I(s)\cdot {P}_{{\rm{Neut}}}(t-s,{x},{y}){ds},$$

in which ${\mathcal{X}}$ denotes the set of all variants present within the time horizon of interest, *P*_Neut_(*t* − *s*, *x*, *y*) denotes the probability that an infection with strain *x*, that occurred *t* − *s* days ago cross-neutralizes a variant *y*. In the equation above, *π*_*x*_(*s*) denotes the proportion of variant *x* at day *s* < *t*, derived from the molecular surveillance, and *I*(*t*) denotes the number of infected individuals at some previous time point *s*. The expected number of susceptible individuals to a variant *y* at time *t* can then be calculated as ${\mathbb{E}}[{{\rm{S}}}_{y}(t)]={\rm{Pop}}-{\mathbb{E}}[{{\rm{Immune}}}_{y}(t)]$, in which Pop denotes the total population size. The variable *I*(*s*) is typically not available, but can be replaced by incidence correlates *ϕ*(*s*) = *I*(*s*)/*c* (see below).

### Variant dynamics

To estimate whether an emerging variant may successfully out-compete existing variants, we estimated the relative growth advantage of a variant *γ*_*y*_(*t*):

$${\gamma }_{y}(t)=\frac{{\alpha }_{y}{\mathbb{E}}[{{\rm{S}}}_{y}(t)]-{\sum }_{x\in {\mathcal{X}}}{\pi }_{x}(t)\cdot {\alpha }_{x}\cdot {\mathbb{E}}[{{\rm{S}}}_{x}(t)]}{{\sum }_{x\in {\mathcal{X}}}{\pi }_{x}(t)\cdot {\alpha }_{x}\cdot {\mathbb{E}}[{{\rm{S}}}_{x}(t)]},$$

in which the denominator denotes the average growth rate across all variants existing at time *t* and for which *α*_*x*_ > 0 denotes a variant’s intrinsic (antibody-independent) relative transmission fitness, which we assumed to be nearly identical for all circulating variants *α*_*x*_ ≈ *α*, implying that variant dynamics are dominated by infection history and immune dynamics. We ignored low-abundance variants with *π*_*x*_(*t*) < 1% and renormalized accordingly. We computed the frequency-weighted average *γ*_*z*_(*t*), whenever different variants were combined during analyses (as indicated in the respective graphics).

### Replacing infection numbers with incidence correlates

**Extended Data Fig. 9:**
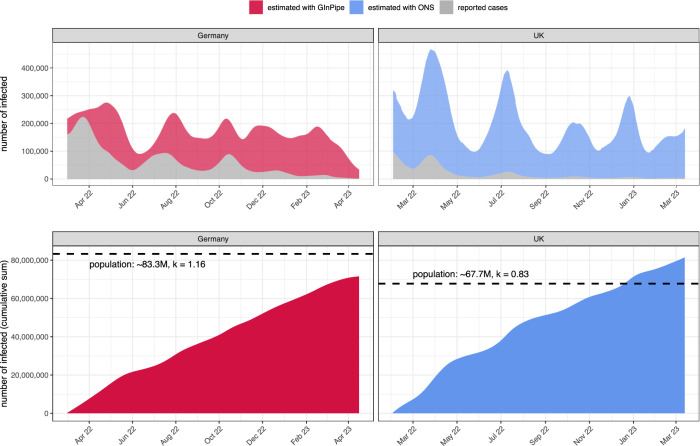
Estimation of the number of infected individuals in Germany (left, red) and the UK (right, blue). The upper panels show the estimated incidence for Germany with GInPipe and for UK using the ONS survey data over a time horizon of 13.5 months (roughly a year). The grey parts of the areas depict the officially reported cases, respectively. In the lower panels, the cumulative sum of the number of infected individuals over time are compared to the population size of the respective country, denoted by the black dashed line. The scaling factor *k* is given by the ratio of the population size and the sum of infected individuals within the time horizon.

**Extended Data Fig. 10:**
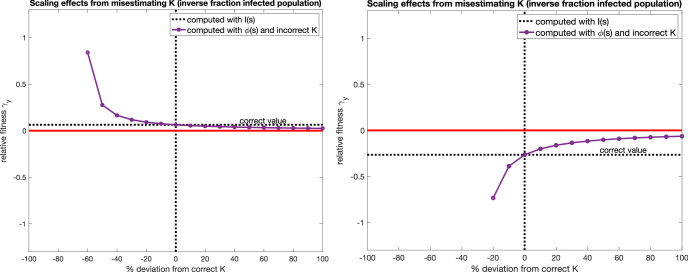
Utilization of incidence correlates. The figure shows how utilization of incidence correlates may affect relative fitness estimates γ_y_ (magenta dots), depending on the choice of *k* (*k*^−1^ = estimate of the expected frequency of infection in the population over the time horizon of interest). Essentially, if *k* is correctly predicted (vertical dashed line), γ_y_ is also correctly estimated with incidence correlates *ϕ(t)* (in comparison to computing γ_y_ with actual infection numbers I(t); solid black horizontal line). If k is over- or under-estimated γ_y_ gets scaled (with a factor ξ). If k is underestimated, the region’s population gets under-estimated, which results in under-estimating the number of susceptibles (over-prediction of competition between variants. Note, that the qualitative results are correct in any case (inflection points γ_y_ = 0; variant ‘growth’ γ_y_ > 0 vs. variant ‘decline’ γ_y_ < 0).

As infection numbers *I*(*t*) are typically unreliable, our method can utilize any incidence correlate, such as GInPipe’s *ϕ*(*t*), wastewater virus load trajectories or reliable estimates from large citizen science projects. With regard to GInPipe’s output, we showed in Extended Data Figs. 3 and 4 and in ref. ^22^ that *I*(*t*) ≈ *cϕ*(*t*), which allows us to use *ϕ*(*s*) instead of the number of infected individuals *I*(*s*) when computing ${\mathbb{E}}[{{\rm{Immune}}}_{x}(t)]$, as performed in Figs. 3–5. To compute ${\mathbb{E}}[{{\rm{S}}}_{x}(t)]$, we can write ${\rm{Pop}}=k\cdot \int I(s){\rm{d}}s\approx k\cdot c\cdot \int \phi (s){\rm{d}}s$, for which *k* > 0 is a scaling factor (for example, *k*^−1^ = 2 implies that every individual got infected twice, on average, over the time horizon of interest). When reconstructing actual incidences for Germany and the UK, we found that *k* ≈ 1 for the length of the time horizon (Extended Data Fig. 9). *k* is a modelling choice and any mis-estimation of *k* affects only the scaling of *γ*_*y*_(*t*) (Extended Data Fig. 10). Qualitative results (inflection points *γ*_*y*_ = 0; variant ‘growth’ *γ*_*y*_ > 0; variant ‘decline’ *γ*_*y*_ < 0) remain unaffected. The interpretation of this ‘scaling’ is straightforward: if one overestimates the number of individuals that became infected, one under-predicts the number of susceptible individuals and thus infers stronger competition among variants, which is expressed in overestimation of the amplitude of *γ*_*y*_(*t*) by some constant *ξ* > 1.

### Relation of *γ*_*y*_ to variant dynamics

In a discrete-time quasi-species model^57^, the theoretical variant frequencies ${\boldsymbol{p}}\in {[\mathrm{0,}1]}^{|{\mathcal{X}}|}$ are given by

$${\boldsymbol{p}}(t+1)={\boldsymbol{Q}}\cdot {\boldsymbol{F}}(t)\cdot {\boldsymbol{p}}(t),$$

for which ${\boldsymbol{Q}}\in {{\mathbb{R}}}^{|{\mathcal{X}}|\times |{\mathcal{X}}|}$ denotes a transition matrix between different variants, which we set to the identity matrix ***Q*** = Id, ignoring any mutational transitions from one variant to another. Fitness values of any variant *y*, relative to the population average $\bar{f}(t)=\,{\sum }_{x}{p}_{x}(t)\cdot {f}_{x}(t)$ are contained in matrix ${\boldsymbol{F}}(t)={\rm{diag}}([\ldots ,\,{f}_{y}(t)/\bar{f}(t),\,\ldots ])$. Consequently, for *p*_*y*_(*t*) > 0, we get

$$\frac{{p}_{y}(t+1)}{{p}_{y}(t)}-1={\gamma }_{y}(t),$$

if fitness is determined by population immunity.

### Modelling booster vaccinations in Germany

As the vaccination timeline contains the number of individuals that were vaccinated, we first reconstructed actual infection numbers for Germany using GInPipe^22^: we inferred the time-dependent case ascertainment probabilities *P*_rep_(*t*) ≤ 1 (that is, the probability of an infection being reported):

$${P}_{{\rm{rep}}}(t)=\frac{{I}_{{\rm{rep}}}(t)}{I(t)}\approx \frac{{I}_{{\rm{rep}}}(t)}{{\phi }_{t}\cdot c}\;\iff \;{P}_{{\rm{rep}}}\cdot c\approx \,\frac{{I}_{{\rm{rep}}}(t)}{{\phi }_{t}},$$

for which *I*_rep_(*t*) ≤ *I*(*t*) denotes the daily reported infections (weekly cases/7). We smoothed reported cases *I*_rep_(*t*) with a bandwidth of 14.

Last, we normalized the case ascertainment probabilities ${\widetilde{P}}_{{\rm{rep}}}(t)$ at maximum to be able to estimate the minimum number of infections *I*_min_(*t*)

$${P}_{{\rm{rep}}}(t)\le {\widetilde{P}}_{{\rm{rep}}}(t)=\frac{{P}_{{\rm{rep}}}(t)}{{\max }_{t}({P}_{{\rm{rep}}}(t))}.$$

The minimum number of infections is then calculated as

$$I(t)\ge {I}_{\min }(t)=\frac{{I}_{{\rm{r}}{\rm{e}}{\rm{p}}}(t)}{{\mathop{P}\limits^{ \sim }}_{{\rm{r}}{\rm{e}}{\rm{p}}}(t)}={\phi }_{t}\mathop{\max }\limits_{t}\,\left(\frac{{I}_{{\rm{r}}{\rm{e}}{\rm{p}}(t)}}{{\phi }_{t}}\right)\ge {I}_{{\rm{r}}{\rm{e}}{\rm{p}}}(t).$$

The impact of infections on the immune landscape was then modelled as outlined above by setting *I*(*t*) ≈ *I*_min_(*t*). To model the impact of booster vaccinations in Germany (Extended Data Fig. 7), we added exposure to either the Wuhan-Hu-1-variant antigen or the BA.4 + BA.5-variant antigen corresponding to the timeline of vaccination (https://impfdashboard.de/en/) to the infection timeline and computed the relative fitness *γ*_*y*_(*t*) on this basis. Note that this reconstruction of actual infection numbers can become unstable, as it is normalized to an extreme point (the maximum) and therefore warrants post-analysis inspection. Moreover, it requires the use of case reporting data, which largely ceased by the end of 2023.

### Reporting summary

Further information on research design is available in the Nature Portfolio Reporting Summary linked to this article.

## Online content

Any methods, additional references, Nature Portfolio reporting summaries, source data, extended data, supplementary information, acknowledgements, peer review information; details of author contributions and competing interests; and statements of data and code availability are available at 10.1038/s41586-024-08477-8.

## Supplementary information

Supplementary Information Supplementary Tables 1–6, containing the definition of epitope classes and their assigned antibodies (Supplementary Tables 1 and 2), spike alteration profiles for vaccine efficacy simulations (Supplementary Table 3), information regarding clinical vaccine efficacies (Supplementary Tables 4 and 5) and a summary for international viral genomics datasets (Supplementary Table 6), and Supplementary Note 1 with GISAID acknowledgements.

Reporting Summary

## Source data

Source Data Fig. 2

Source Data Fig. 3

Source Data Fig. 4

Source Data Fig. 5

## Data Availability

The DMS data used in this study can be accessed via GitHub at https://github.com/jbloomlab/SARS2_RBD_Ab_escape_maps/blob/main/processed_data/escape_data.csv and a processed version is available via GitHub at https://github.com/KleistLab/VASIL/blob/main/ByCountry/Australia/results/epitope_data/dms_per_ab_per_site.csv. For the evaluation of the German SARS-CoV-2 outbreak, we used genomic data provided via the German Sequence Data Hub (DESH) to the Robert Koch Institute, available via GitHub at https://github.com/robert-koch-institut/SARS-CoV-2-Sequenzdaten_aus_Deutschland, and via Zenodo at https://zenodo.org/records/13987397 (ref. ^58^; details for the subset in Supplementary Table 6). Wastewater surveillance data for Germany are provided by the Robert Koch Institute via GitHub at https://github.com/robert-koch-institut/Abwassersurveillance_AMELAG and can be accessed via Zenodo at https://zenodo.org/records/12704658 (ref. ^59^). Reported case numbers for Germany were taken from GitHub at https://github.com/robert-koch-institut/COVID-19_7-Tage-Inzidenz_in_Deutschland. The evaluation of other countries in this study was based on genomic data associated with 5,617,986 SARS-CoV-2 sequences available on GISAID (https://gisaid.org/) and accessible at 10.55876/gis8.241022rp (Supplementary Note 1 and Supplementary Table 6). Source data are provided with this paper.

## Code availability

Codes were written in Python 3.11.3 and R version 4.2.3 (15 March 2023) and are available via GitHub at https://github.com/KleistLab/VASIL and via Zenodo at 10.5281/zenodo.8349295 (ref. ^60^). Simulations were performed on the high-performance computing cluster at ZEDAT, Freie Universität Berlin^61^. The pipeline for the genome-based incidence estimation (GInPipe) is available via GitHub at https://github.com/KleistLab/GInPipe. Alteration profiles for all sequences were extracted using covSonar, version 1.1.11, which is available via GitHub at https://github.com/rki-mf1/covsonar.
