# Supplementary material for: SARS-CoV-2 evolution on a dynamic immune landscape
Source: Nature. 2025 Jan 29;639(8053):196–204. doi: 10.1038/s41586-024-08477-8 (PMC11882442; doi:10.1038/s41586-024-08477-8)
Supplement: Supplementary file 1 — Supplementary Tables 1–6, containing the definition of epitope classes and their assigned antibodies (Supplementary Tables 1 and 2), spike alteration profiles for vaccine efficacy simulations (Supplementary Table 3), information regarding clinical vaccine efficacies (Supplementary Tables 4 and 5) and a summary for international viral genomics datasets (Supplementary Table 6), and Supplementary Note 1 with GISAID acknowledgements. [file 41586_2024_8477_MOESM1_ESM.pdf]

nature portfolio

https://doi.org/10.1038/s41586-024-08477-8

Supplementary information

SARS-CoV-2 evolution on a dynamic immune landscape

In the format provided by the authors and unedited

Supplementary Information for:
SARS-CoV-2 Evolution on a Dynamic Immune Landscape

N. Alexia Raharinirina[1,*], Nils Gubela[1,2,*], Daniela Börnigen[3,*], Maureen Rebecca Smith[3,*], Djin-Ye Oh[4], Matthias Budt[4], Christin Mache[4], Claudia Schillings[1], Stephan Fuchs[5], Ralf Dürrwald[4], Thorsten Wolff[4], Martin Hölzer[5], Sofia Paraskevopoulou[5], and Max von Kleist[1,3,+]
[1] Department of Mathematics & Computer Science, Freie UniversitÅNat Berlin, Germany
[2] International Max-Planck Research School "Biology and Computation" (IMPRS-BAC), Max-Planck Institute for Molecular Genetics, Berlin, Germany
[3] Project groups, Robert-Koch Institute, Berlin, Germany
[4] Department 1, Robert-Koch Institute, Berlin, Germany
[5] Department MFI, Robert-Koch Institute, Berlin, Germany

*these authors contributed equally
+max.kleist@fu-berlin.de

Table of Contents

Supplementary Table 1: Number of antibodies per epitope class. **3**

Supplementary Table 2: List of antibodies per epitope class...**4**

Supplementary Table 4: Vaccine efficacy data against Delta as extracted from the literature. **22**

Supplementary Table 5: Vaccine efficacy data against Omicron as extracted from the literature. **23**

Supplementary Table 6: Summary of genomic surveillance data sets across all 12 investigated countries...**24**

Supplementary Note 1: Acknowledgements for GISAID sequences. **25**

Supplementary Table 1: Number of antibodies per epitope class.

Epitope Class	Number of antibodies
A	109
B	51
C	88
D1	40
D2	32
E1	61
E2.1	25
E2.2	41
E3	63
F1	92
F2	167
F3	67

Supplementary Table 2: List of antibodies per epitope class.

Epitope Class	Antibody
A	B38
A	BD-236
A	BD-369
A	BD-494
A	BD-498
A	BD-500
A	BD-503
A	BD-504
A	BD-508
A	BD-598
A	BD-599
A	BD-605
A	BD-612
A	BD-614
A	BD-616
A	BD-618
A	BD-619
A	BD-693
A	BD-694
A	BD-715
A	BD-739
A	BD-822
A	BD-915
A	BD55-1061
A	BD55-1092
A	BD55-1208
A	BD55-1249
A	BD55-1315
A	BD55-1346
A	BD55-1374
A	BD55-1451
A	BD55-1456
A	BD55-1502
A	BD55-1503
A	BD55-1575
A	BD55-1763
A	BD55-1809
A	BD55-1970
A	BD55-300
A	BD55-5580
A	BD55-5731
A	BD55-5743
A	BD55-5856
A	BD55-5966
A	BD55-600
A	BD55-6413
A	BG4-25

A	BG7-20
A	BRII-196
A	C093
A	C102
A	C105
A	C135
A	C1A-B12
A	C1A-B3
A	C1A-C2
A	C1A-F10
A	C578
A	CC12.1
A	CC12.3
A	COV2-2037
A	COV2-2068
A	COV2-2098
A	COV2-2113
A	COV2-2308
A	COV2-2589
A	COV2-2807
A	COV2-2919
A	COVA2-04
A	COVOX-150
A	COVOX-222
A	COVOX-269
A	COVOX-40
A	COVOX-88
A	CV05-163
A	CV07-250
A	CV30
A	DH1126
A	DH1140
A	DH1154
A	DH1155
A	DH1179
A	DH1210
A	DXP-604
A	Ehling_mAb-64
A	LY-CoV016
A	LY-CoV481
A	LY-CoV488
A	P2B-1A10
A	P4A1
A	P5A-2G9
A	P5A-3A1
A	WIBP-2B11
A	XG2v-039
A	XG2v-098
A	XGv-013
A	XGv-017
A	XGv-040

A	XGv-177
A	XGv-208
A	XGv-225
A	XGv-261
A	XGv-298
A	XGv-300
A	XGv-301
A	XGv-304
A	XGv-387
A	XGv-404
A	XGv-405
B	BD-319
B	BD-417
B	BD-566
B	BD-623
B	BD-805
B	BD-833
B	BD-836
B	BD-922
B	BD55-1180
B	BD55-1263
B	BD55-1334
B	BD55-1421
B	BD55-1508
B	BD55-4662
B	BD55-5183
B	BD55-5324
B	BD55-5399
B	BD55-5463
B	BD55-5530
B	BD55-556
B	BD55-5655
B	BD55-5745
B	BD55-5774
B	BD55-5811
B	BD55-6382
B	BD55-6383
B	BD56-887
B	BG1-24
B	C597
B	COV2-2046
B	COV2-2072
B	COV2-2196
B	COV2-2381
B	COV2-2684
B	COV2-2941
B	COV2-3025
B	COVA2-39
B	COVOX-253H165L
B	COVOX-253H55L
B	CV-X2-106

B	CV07-209
B	CV07-287
B	S2E12
B	XG2v-095
B	XG2v-126
B	XGv-050
B	XGv-253
B	XGv-279
B	XGv-347
B	XGv-402
B	XGv-409
C	2-15
C	BD-254
C	BD-362
C	BD-368
C	BD-397
C	BD-403
C	BD-536
C	BD-790
C	BD-791
C	BD-870
C	BD-900
C	BD55-1049
C	BD55-1051
C	BD55-1065
C	BD55-1078
C	BD55-1084
C	BD55-1171
C	BD55-1192
C	BD55-1223
C	BD55-1256
C	BD55-1323
C	BD55-1435
C	BD55-1558
C	BD55-1576
C	BD55-1579
C	BD55-1694
C	BD55-4351
C	BD55-4382
C	BD55-4397
C	BD55-5634
C	BD55-5747
C	BD55-5854
C	BD55-5909
C	C002
C	C058
C	C083
C	C085
C	C121
C	C144
C	C516

C	C591
C	CM32
C	COV2-2064
C	COV2-2353
C	COV2-2391
C	COV2-2479
C	COV2-2504
C	COV2-2539
C	COV2-2819
C	COV2-2955
C	COVOX-316
C	COVOX-384
C	CV07-200
C	CV07-222
C	CV07-255
C	CV07-262
C	CV07-283
C	CV07-315
C	DH1041
C	DH1042
C	DH1043
C	DH1082
C	DH1111
C	DH1143
C	DH1159-2
C	DH1173
C	DH1184
C	DH1186
C	DH1196
C	DH1214
C	DXP-593
C	H4
C	LY-CoV555
C	P2C-1A3
C	S2H13
C	S2M11
C	XG2v-045
C	XGv-051
C	XGv-052
C	XGv-055
C	XGv-097
C	XGv-152
C	XGv-155
C	XGv-172
C	XGv-232
C	XGv-324
C	XGv-337
C	XGv-343
D1	1-57
D1	BD-643
D1	BD-804

D1	BD-824
D1	BD-864
D1	BD-918
D1	BD55-1009
D1	BD55-1039
D1	BD55-1268
D1	BD55-1354
D1	BD55-1706
D1	BD55-1779
D1	BD55-1962
D1	BD55-5462
D1	BD55-5566
D1	BD55-5721
D1	BD55-5836
D1	BD55-6385
D1	C110
D1	C119
D1	COV2-2130
D1	COV2-2693
D1	COV2-2780
D1	COVOX-75
D1	CV07-270
D1	DH1044
D1	DH1160
D1	DH1161-2
D1	Ehling_mAb-82
D1	P2B-2F6
D1	TAU-2230
D1	XG2v-052
D1	XG2v-083
D1	XGv-010
D1	XGv-047
D1	XGv-191
D1	XGv-250
D1	XGv-263
D1	XGv-345
D1	XGv-420
D2	BD-467
D2	BD-812
D2	BD-817
D2	BD55-1104
D2	BD55-1109
D2	BD55-1136
D2	BD55-387
D2	BG7-15
D2	COV2-2268
D2	COV2-2499
D2	Ehling_mAb-50
D2	LY-CoV1404
D2	XGv-016
D2	XGv-074

D2	XGv-107
D2	XGv-264
D2	XGv-265
D2	XGv-266
D2	XGv-282
D2	XGv-285
D2	XGv-286
D2	XGv-287
D2	XGv-288
D2	XGv-289
D2	XGv-290
D2	XGv-291
D2	XGv-292
D2	XGv-293
D2	XGv-294
D2	XGv-295
D2	XGv-296
D2	XGv-297
E1	BD-907
E1	BD55-1091
E1	BD55-1114
E1	BD55-1232
E1	BD55-1245
E1	BD55-1294
E1	BD55-1339
E1	BD55-1403
E1	BD55-2532
E1	BD55-3149
E1	BD55-3152
E1	BD55-3433
E1	BD55-3440
E1	BD55-3451
E1	BD55-3457
E1	BD55-3546
E1	BD55-3637
E1	BD55-4281
E1	BD55-4285
E1	BD55-4325
E1	BD55-4484
E1	BD55-4495
E1	BD55-4664
E1	BD55-4892
E1	BD55-5001
E1	BD55-5171
E1	BD55-5175
E1	BD55-5195
E1	BD55-5200
E1	BD55-5219
E1	BD55-5228
E1	BD55-5286
E1	BD55-5303

E1	BD55-5319
E1	BD55-5382
E1	BD55-5386
E1	BD55-5449
E1	BD55-5473
E1	BD55-5477
E1	BD55-5484
E1	BD55-5501
E1	BD55-5542
E1	BD55-5549
E1	BD55-5565
E1	BD55-5585
E1	BD55-5591
E1	BD55-5612
E1	BD55-5643
E1	BD55-5644
E1	BD55-5697
E1	BD55-5718
E1	BD55-5728
E1	BD55-5779
E1	BD55-5832
E1	BD55-5840
E1	BD55-6241
E1	BG10-19
E1	C576
E1	CV38-142
E1	S309
E1	XGv-421
E2.1	BD-713
E2.1	BD-744
E2.1	BD-914
E2.1	BD-923
E2.1	BD55-1029
E2.1	BD55-1073
E2.1	BD55-1274
E2.1	BD55-1312
E2.1	BD55-1432
E2.1	BD55-302
E2.1	BD55-311
E2.1	C556
E2.1	COV2-2389
E2.1	DH1151
E2.1	XG2v-070
E2.1	XGv-011
E2.1	XGv-094
E2.1	XGv-174
E2.1	XGv-175
E2.1	XGv-199
E2.1	XGv-214
E2.1	XGv-227
E2.1	XGv-233

E2.1	XGv-284
E2.1	XGv-346
E2.2	BD-692
E2.2	BD-748
E2.2	BD-796
E2.2	BD-913
E2.2	BD55-1251
E2.2	BD55-1266
E2.2	BD55-1275
E2.2	BD55-1307
E2.2	BD55-1442
E2.2	BD55-1472
E2.2	BD55-1831
E2.2	BD55-1957
E2.2	BD55-3300
E2.2	BD55-3370
E2.2	BD55-3484
E2.2	BD55-3525
E2.2	BD55-3698
E2.2	BD55-4467
E2.2	BD55-5320
E2.2	BD55-5413
E2.2	BD55-5524
E2.2	BD55-5598
E2.2	BD55-5617
E2.2	BD55-5789
E2.2	BD55-5844
E2.2	BD55-6078
E2.2	BD55-6511
E2.2	BD55-6637
E2.2	C091
E2.2	C581
E2.2	COV2-2485
E2.2	DH1193
E2.2	XG2v-006
E2.2	XGv-030
E2.2	XGv-038
E2.2	XGv-072
E2.2	XGv-198
E2.2	XGv-20
E2.2	XGv-212
E2.2	XGv-251
E2.2	XGv-380
E3	BD-815
E3	BD55-1027
E3	BD55-1041
E3	BD55-1140
E3	BD55-1146
E3	BD55-1199
E3	BD55-1241
E3	BD55-1405

E3	BD55-1453
E3	BD55-1458
E3	BD55-1486
E3	BD55-1570
E3	BD55-1811
E3	BD55-2314
E3	BD55-3150
E3	BD55-3327
E3	BD55-3337
E3	BD55-3417
E3	BD55-3561
E3	BD55-3597
E3	BD55-3611
E3	BD55-3773
E3	BD55-3807
E3	BD55-3832
E3	BD55-4804
E3	BD55-5212
E3	BD55-5251
E3	BD55-5263
E3	BD55-5268
E3	BD55-5293
E3	BD55-5379
E3	BD55-5415
E3	BD55-5418
E3	BD55-5470
E3	BD55-5485
E3	BD55-5545
E3	BD55-5551
E3	BD55-5569
E3	BD55-5583
E3	BD55-5625
E3	BD55-5630
E3	BD55-5687
E3	BD55-5752
E3	BD55-5830
E3	BD55-5858
E3	BD55-5992
E3	BD55-6041
E3	BD55-6234
E3	BD55-6346
E3	BD55-6585
E3	BD55-749
E3	BD55-751
E3	COVA1-16
E3	DH1072
E3	S2H97
E3	TAU-2310
E3	XGv-014
E3	XGv-179
E3	XGv-235

E3	XGv-355
E3	XGv-401
E3	XGv-418
E3	XGv-422
F1	BD-708
F1	BD55-1004
F1	BD55-1038
F1	BD55-1064
F1	BD55-1070
F1	BD55-1128
F1	BD55-1132
F1	BD55-1226
F1	BD55-1242
F1	BD55-1244
F1	BD55-1297
F1	BD55-1338
F1	BD55-1341
F1	BD55-1398
F1	BD55-1416
F1	BD55-1429
F1	BD55-2413
F1	BD55-3161
F1	BD55-3293
F1	BD55-3324
F1	BD55-3366
F1	BD55-3602
F1	BD55-3684
F1	BD55-3741
F1	BD55-4286
F1	BD55-4328
F1	BD55-4329
F1	BD55-4342
F1	BD55-4360
F1	BD55-4364
F1	BD55-4392
F1	BD55-4394
F1	BD55-4418
F1	BD55-4464
F1	BD55-5215
F1	BD55-5248
F1	BD55-5250
F1	BD55-5271
F1	BD55-5284
F1	BD55-5311
F1	BD55-5326
F1	BD55-5358
F1	BD55-5359
F1	BD55-5361
F1	BD55-5400
F1	BD55-5404
F1	BD55-5417

F1	BD55-5425
F1	BD55-5442
F1	BD55-5453
F1	BD55-5454
F1	BD55-5456
F1	BD55-5471
F1	BD55-5476
F1	BD55-5495
F1	BD55-5502
F1	BD55-5557
F1	BD55-5568
F1	BD55-5575
F1	BD55-5577
F1	BD55-5579
F1	BD55-5609
F1	BD55-5636
F1	BD55-5770
F1	BD55-5784
F1	BD55-5790
F1	BD55-5794
F1	BD55-5815
F1	BD55-5879
F1	BD55-5889
F1	BD55-5900
F1	BD55-5961
F1	BD55-6034
F1	BD55-6037
F1	BD55-6038
F1	BD55-6055
F1	BD55-6075
F1	BD55-6222
F1	BD55-746
F1	S304
F1	XGv-001
F1	XGv-018
F1	XGv-023
F1	XGv-024
F1	XGv-049
F1	XGv-073
F1	XGv-19
F1	XGv-252
F1	XGv-305
F1	XGv-306
F1	XGv-311
F1	XGv-318
F2	BD-702
F2	BD-801
F2	BD-899
F2	BD55-1075
F2	BD55-1096
F2	BD55-1117

F2	BD55-1129
F2	BD55-1225
F2	BD55-1233
F2	BD55-1237
F2	BD55-1239
F2	BD55-1273
F2	BD55-1343
F2	BD55-1411
F2	BD55-1412
F2	BD55-1868
F2	BD55-2696
F2	BD55-316
F2	BD55-3304
F2	BD55-347
F2	BD55-3500
F2	BD55-3670
F2	BD55-3716
F2	BD55-3721
F2	BD55-389
F2	BD55-4343
F2	BD55-4344
F2	BD55-4345
F2	BD55-4348
F2	BD55-4356
F2	BD55-4357
F2	BD55-4358
F2	BD55-4483
F2	BD55-4760
F2	BD55-5164
F2	BD55-5167
F2	BD55-5170
F2	BD55-5182
F2	BD55-5189
F2	BD55-5201
F2	BD55-5223
F2	BD55-5226
F2	BD55-5236
F2	BD55-5238
F2	BD55-5239
F2	BD55-5242
F2	BD55-5245
F2	BD55-5256
F2	BD55-5264
F2	BD55-5265
F2	BD55-5267
F2	BD55-5276
F2	BD55-5296
F2	BD55-5301
F2	BD55-5304
F2	BD55-5309
F2	BD55-5333

F2	BD55-5339
F2	BD55-5343
F2	BD55-5380
F2	BD55-5384
F2	BD55-5387
F2	BD55-5397
F2	BD55-5411
F2	BD55-5416
F2	BD55-5419
F2	BD55-5427
F2	BD55-5431
F2	BD55-5432
F2	BD55-5438
F2	BD55-5450
F2	BD55-5467
F2	BD55-547
F2	BD55-5474
F2	BD55-5486
F2	BD55-5493
F2	BD55-5505
F2	BD55-5512
F2	BD55-5516
F2	BD55-5517
F2	BD55-5521
F2	BD55-5537
F2	BD55-5564
F2	BD55-5594
F2	BD55-5610
F2	BD55-5620
F2	BD55-5638
F2	BD55-5640
F2	BD55-5662
F2	BD55-5675
F2	BD55-5676
F2	BD55-5690
F2	BD55-5694
F2	BD55-5699
F2	BD55-5700
F2	BD55-5736
F2	BD55-5737
F2	BD55-5792
F2	BD55-5831
F2	BD55-5839
F2	BD55-5874
F2	BD55-5878
F2	BD55-5927
F2	BD55-6015
F2	BD55-6030
F2	BD55-6033
F2	BD55-6063
F2	BD55-6066

F2	BD55-6068
F2	BD55-6080
F2	BD55-6086
F2	BD55-6088
F2	BD55-6092
F2	BD55-6095
F2	BD55-6103
F2	BD55-6105
F2	BD55-6190
F2	BD55-6197
F2	BD55-6231
F2	BD55-6243
F2	BD55-6251
F2	BD55-6262
F2	BD55-6263
F2	BD55-6283
F2	BD55-6305
F2	BD55-6306
F2	BD55-6308
F2	BD55-6317
F2	BD55-632
F2	BD55-6379
F2	BD55-6392
F2	BD55-6486
F2	BD55-6507
F2	BD55-6510
F2	BD55-6564
F2	BD55-659
F2	BD55-6642
F2	BD55-6647
F2	BD55-676
F2	BD55-688
F2	BD55-711
F2	BD55-747
F2	BD55-822
F2	BD55-885
F2	BD55-948
F2	C048
F2	C594
F2	COV2-2015
F2	COV2-2103
F2	COV2-2258
F2	COV2-2514
F2	COV2-2531
F2	COV2-2677
F2	COV2-2678
F2	COV2-2828
F2	COV2-2841
F2	DH1045
F2	DH1046
F2	DH1047

F2	DH1073
F2	S2X259
F2	XGv-032
F2	XGv-157
F2	XGv-158
F2	XGv-159
F2	XGv-236
F2	XGv-416
F3	ADG-2
F3	BD-449
F3	BD55-1450
F3	BD55-1789
F3	BD55-3372
F3	BD55-3414
F3	BD55-4637
F3	BD55-5163
F3	BD55-5168
F3	BD55-5196
F3	BD55-5198
F3	BD55-5233
F3	BD55-5259
F3	BD55-5300
F3	BD55-5305
F3	BD55-5323
F3	BD55-5325
F3	BD55-5342
F3	BD55-5354
F3	BD55-5365
F3	BD55-5396
F3	BD55-5408
F3	BD55-5448
F3	BD55-5459
F3	BD55-5466
F3	BD55-5468
F3	BD55-5472
F3	BD55-5483
F3	BD55-5514
F3	BD55-5526
F3	BD55-5543
F3	BD55-5558
F3	BD55-5588
F3	BD55-5593
F3	BD55-5615
F3	BD55-5629
F3	BD55-5649
F3	BD55-5713
F3	BD55-5715
F3	BD55-5761
F3	BD55-5785
F3	BD55-5810
F3	BD55-5906

F3	BD55-6025
F3	BD55-6069
F3	BD55-6112
F3	BD55-6195
F3	BD55-6203
F3	BD55-6206
F3	BD55-6208
F3	BD55-6261
F3	BD55-6329
F3	BD55-6340
F3	BD55-6411
F3	BD55-6447
F3	BD55-6477
F3	BD55-6478
F3	BD55-6520
F3	BD55-6527
F3	BD55-6552
F3	BD55-6626
F3	BD55-6638
F3	BD55-6651
F3	S2H14
F3	S2X35
F3	XGv-116
F3	XGv-360

Supplementary Table 3: Omicron (BA.1) and Delta spike mutation profiles for vaccine efficacy simulations.

Mutations in the spike protein of Omicron and Delta lineages as observed between November 20, 2021 until January 31, 2022 (for studies evaluating Wuhan-strain vaccine efficacy against Omicron) and July 4, 2021 until December 31, 2021 (for studies evaluating Wuhan-strain vaccine efficacy against Delta).

**Omicron Spike mutations**	**Delta Spike mutations**
A67V	D614G
D614G	D950N
D796Y	G142D
E484A	L452R
G339D	P681R
G496S	T19R
H655Y	T478K
L981F	
N501Y	
N679K	
N764K	
N856K	
N969K	
P681H	
Q493R	
Q498R	
Q954H	
S371L	
S373P	
S375F	
S477N	
T478K	
T547K	
T95I	
Y505H	

Supplementary Table 4: Vaccine efficacy data against Delta as extracted from the literature.

Source	Days (ranges)	Vaccine Efficacy (%)	CI (%)	Vaccine	Disease Status	Study Region	Study Design	Used Statistic
**Hansen et al. 2022** (10.1101/2021.12.20.21267966)	1 - 30	86.7	84.6 – 88.6	Pfizer	Any infection	Denmark	Test-negative case-control	Hazard Ratio
	31 – 60	80.9	79 – 82.6					
	61 – 90	72.8	71.7 – 73.8					
	91 – 150	53.8	52.9 – 54.6					
	1 – 30	88.2	83.1 – 91.8	Moderna				
	31 – 60	81.5	77.7 – 84.6					
	61 – 90	72.2	70.4 – 74					
	91 – 150	65	63.6 – 66.3					
**Gram et al. 2022** (10.1371/journal.pmed.1003992)	14 – 30	92.2	91.8 – 92.6	Moderna/Pfizer (18-59 yo)	Any infection	Denmark	Test-negative case-control	Hazard Ratio
	31 – 60	88.1	87.7 – 88.5					
	61 – 90	80.8	80.2 – 81.2					
	91 – 120	72.2	71.5 – 72.8					
	120 – 149	64.8	63.9 – 65.8					
	14 – 30	82.3	75.5 – 87.2	Moderna/Pfizer (<60 yo)				
	31 – 60	74.4	70.1 – 78.2					
	61 – 90	77.3	74.4 – 79.9					
	91 – 120	69.6	66.5 – 72.4					
	120 – 149	50	46.7 – 53					
**Tartof et al. 2021** (10.1016/S0140-6736(21)02183-8)	28 – 35	92.423	85.27 – 98.13	Pfizer	Any infection	USA	Retrospective cohort	Hazard Ratio
	56 – 63	87.141	81.3 – 92.08					
	84 – 91	76.936	70.14 – 83.19					
	112 – 119	59.191	48.20 – 69.19					
	140 – 147	52.525	39.12 – 65.22					
**Rev. Feikin et al.2022 (Goldberg et al. 2021)** (10.1016/S0140-6736(22)00152-0 (10.1056/NEJMoa2114228))	41 – 91	82	70 – 89	Pfizer	Any infection	Israel	Retrospective cohort	Rate of confirmed infection
	72 – 121	81	73 – 86					
	102 – 137	73	67 – 79					
	118 – 152	74	68 – 79					
	133 – 166	67	59 – 73					
	147 – 180	63	58 – 67					
	161 – 196	57	52 – 62					
**Rev. Feikin et al.2022 (Poukka al. 2020)** (10.1016/S0140-6736(22)00152-0 (10.1016/j.vaccine.2021.12.032))	14 – 90	88	71 – 95	Astrazeneca	Any infection	Finland	Retrospective cohort	Hazard Ratio
	91 – 180	62	17 – 95					

Supplementary Table 5: Vaccine efficacy data against Omicron as extracted from the literature.

Source	Days (ranges)	Vaccine Efficacy (%)	CI (%)	Vaccine	Disease Status	Study Region	Study Design	Used Statistic
**Hansen et al. 2022** (10.1101/2021.12.20.21267966)	1 – 30	55.2	23.5 – 73.7	Pfizer	Any infection	Denmark	Test-negative case-control	Hazard Ratio
	31 – 60	16.1	(-20.8) – 41.7					
	61 – 90	9.8	(-10) – 26.1					
	1 – 30	36.7	(-69.9) – 76.4	Moderna				
	31 – 60	30	(-41.3) – 65.4					
	61 – 90	4	(-30.8) – 29.8					
**Gram et al. 2022** (10.1371/journal.pmed.1003992)	14 – 30	40	38.6 – 41.3	Moderna/Pfizer(18-59 yo)	Any infection	Denmark	Test-negative case-control	Hazard Ratio
	31 – 60	31.9	30.7 – 33					
	61 – 90	32.3	30.9 – 33.7					
	91 – 120	31.3	30.3 – 32.4					
	120 – 149	12.6	12 – 13.3					
	14 – 30	39.9	26.3 – 50.9	Moderna/Pfizer (<60 yo)				
	31 – 60	39	27.6 – 48.7					
	61 – 90	25.2	9 – 38.6					
	91 – 120	24	11.4 – 34.8					
	120 – 149	4.4	(-0.1) – 8.7					

Supplementary Table 6: Summary of genomic surveillance data sets across all 12 investigated countries.

Country	Data time-horizon	Prediction time-horizon	Number of valid genomes	Minimun Sequences per time-steps	Number of lineages	Number of lineages in Spike-pseudo-groups	Number of Spike-pseudo-groups (> 1%)
Germany	2021-07-01 – 2024-07-01	2022-03-01 – 2024-07-01	607798	100	1718	756	227
USA	2022-02-01 – 2023-11-27	2022-10-01 – 2023-11-20	1958675	100	1736	1062	86
United Kingdom	2022-02-01 – 2023-11-23	2022-10-01 – 2023-11-15	975875	100	1468	1127	165
Australia	2022-02-01 – 2023-11-23	2022-10-01 – 2023-11-16	151453	100	1242	947	201
Brazil	2022-02-01 – 2023-11-24	2022-10-01 – 2023-10-30	87212	100	501	302	118
Canada	2022-02-01 – 2023-11-24	2022-10-01 – 2023-11-16	284354	100	1312	1030	141
Sweden	2022-02-01 – 2023-11-24	2022-10-01 – 2023-11-17	101188	100	1010	753	239
France	2022-02-01 – 2023-11-25	2022-10-01 – 2023-11-20	311205	100	1347	1018	155
Denmark	2022-02-01 – 2023-11-20	2022-10-01 – 2023-11-16	296003	100	1134	835	130
Mexico	2022-02-01 – 2023-10-17	2022-10-01 – 2023-09-14	35141	100	556	409	127
Japan	2022-02-01 – 2023-11-17	2022-10-01 – 2023-11-10	403515	100	1192	890	141
South Africa	2022-02-01 – 2023-11-09	2022-10-01 – 2023-09-20	17152	100	411	296	120

Supplementary Note 1: Acknowledgements for GISAID sequences.

SUPPLEMENTAL TABLE

Data Availability

GISAID Identifier:	EPI_SET_241022rp
doi:	10.55876/gis8.241022rp

All genome sequences and associated metadata in this dataset are published in GISAID's EpiCoV database. To view the contributors of each individual sequence with details such as accession number, Virus name, Collection date, Originating Lab and Submitting Lab and the list of Authors, visit 10.55876/gis8.241022rp

Data Snapshot

- EPI_SET_241022rp is composed of 5,617,986 individual genome sequences.
- The collection dates range from 2020-07-06 to 2023-11-27;
- Data were collected in 16 countries and territories;
- All sequences in this dataset are compared relative to hCoV-19/Wuhan/WIV04/2019 (WIV04), the official reference sequence employed by GISAID (EPI_ISL_402124). Learn more at https://gisaid.org/WIV04.
