# Supplementary material for: SARS-CoV-2 evolution on a dynamic immune landscape
Source: Nature. 2025 Jan 29;639(8053):196–204. doi: 10.1038/s41586-024-08477-8 (PMC11882442; doi:10.1038/s41586-024-08477-8)
Supplement: Supplementary file 2 — Reporting Summary [file 41586_2024_8477_MOESM2_ESM.pdf]

nature portfolio

Corresponding author(s): Max von Kleist

Last updated by author(s): Oct 31, 2024

Reporting Summary

Nature Portfolio wishes to improve the reproducibility of the work that we publish. This form provides structure for consistency and transparency in reporting. For further information on Nature Portfolio policies, see our Editorial Policies and the Editorial Policy Checklist.

Statistics

For all statistical analyses, confirm that the following items are present in the figure legend, table legend, main text, or Methods section.

n/a	Confirmed	
☐	☒	The exact sample size (*n*) for each experimental group/condition, given as a discrete number and unit of measurement
☒	☐	A statement on whether measurements were taken from distinct samples or whether the same sample was measured repeatedly
☐	☒	The statistical test(s) used AND whether they are one- or two-sided<br>*Only common tests should be described solely by name; describe more complex techniques in the Methods section.*
☒	☐	A description of all covariates tested
☒	☐	A description of any assumptions or corrections, such as tests of normality and adjustment for multiple comparisons
☐	☒	A full description of the statistical parameters including central tendency (e.g. means) or other basic estimates (e.g. regression coefficient) AND variation (e.g. standard deviation) or associated estimates of uncertainty (e.g. confidence intervals)
☐	☒	For null hypothesis testing, the test statistic (e.g. *F*, *t*, *r*) with confidence intervals, effect sizes, degrees of freedom and *P* value noted<br>*Give P values as exact values whenever suitable.*
☒	☐	For Bayesian analysis, information on the choice of priors and Markov chain Monte Carlo settings
☒	☐	For hierarchical and complex designs, identification of the appropriate level for tests and full reporting of outcomes
☒	☐	Estimates of effect sizes (e.g. Cohen's *d*, Pearson's *r*), indicating how they were calculated

Our web collection on statistics for biologists contains articles on many of the points above.

Software and code

Policy information about availability of computer code

Data collection	Data processing is described in Method section. No software was used for data extraction.
Data analysis	We used custom codes implemented in Python version 3.11.3. and R version 4.2.3 and are available at https://github.com/KleistLab/VASIL, including an installation guide. Codes used in the analysis (VASIL version 2.0) were deposited DOI: 10.5281/zenodo.10572808. A one-click capsule at CodeOcean.com is available at doi: 10.24433/CO.8711362.v1. The pipeline for the genome-based incidence estimation (GInPipe) is available at https://github.com/KleistLab/GInPipe, version 3.0.0. Mutation profiles for all sequences were extracted using covSonar, version 1.1.11 (https://github.com/rki-mf1/covsonar)

For manuscripts utilizing custom algorithms or software that are central to the research but not yet described in published literature, software must be made available to editors and reviewers. We strongly encourage code deposition in a community repository (e.g. GitHub). See the Nature Portfolio guidelines for submitting code & software for further information.

Data

Policy information about availability of data

All manuscripts must include a data availability statement. This statement should provide the following information, where applicable:

- Accession codes, unique identifiers, or web links for publicly available datasets
- A description of any restrictions on data availability
- For clinical datasets or third party data, please ensure that the statement adheres to our policy

All original data is publicly available. processed data is provided through the project's Github (zenodo and CodeOcean). The DMS data used in this study can be accessed at https://github.com/jbloomlab/SARS2_RBD_Ab_escape_maps/blob/main/processed_data/escape_data.csv and a processed version can be found at https://github.com/KleistLab/VASIL/blob/main/ByCountry/Australia/results/epitope_data/dms_per_ab_per_site.csv.
For the evaluation of the German SARS-CoV-2 outbreak we used genomic data provided via the German Sequence Data Hub (DESH) to the Robert Koch Institute https://github.com/robert-koch-institut/SARS-CoV-2-Sequenzdaten_aus_Deutschland, accessible at zenodo https://zenodo.org/records/13987397 f (details for the subset in Suppl. Table 6).
Wastewater surveillance data for Germany is provided by the Robert Koch Institute https://github.com/robert-koch-institut/Abwassersurveillance_AMELAG and can be accessed via zenodo https://zenodo.org/records/12704658. Reported case numbers for Germany were taken from https://github.com/robert-koch-institut/COVID-19_7-Tage-Inzidenz_in_Deutschland.
The evaluation of other countries (Fig. 4) in this study was based on genomic data associated with 5,617,986 SARS-CoV-2 sequences available on GISAID (https://gisaid.org/) and accessible at https://doi.org/10.55876/gis8.241022rp (Suppl. Note 1 and Suppl. Table 6).

Research involving human participants, their data, or biological material

Policy information about studies with human participants or human data. See also policy information about sex, gender (identity/presentation), and sexual orientation and race, ethnicity and racism.

Reporting on sex and gender	N/A. This research does not involve human research participants
Reporting on race, ethnicity, or other socially relevant groupings	N/A. The research does not take into account race, ethnicity, or any other socially relevant groupings.
Population characteristics	N/A. Population characteristics is not relevant here.
Recruitment	N/A. Recruitment is not relevant here.
Ethics oversight	N/A. Ethics is not relevant here.

Note that full information on the approval of the study protocol must also be provided in the manuscript.

Field-specific reporting

Please select the one below that is the best fit for your research. If you are not sure, read the appropriate sections before making your selection.

☒ Life sciences ☐ Behavioural & social sciences ☐ Ecological, evolutionary & environmental sciences

For a reference copy of the document with all sections, see nature.com/documents/nr-reporting-summary-flat.pdf

Life sciences study design

All studies must disclose on these points even when the disclosure is negative.

Sample size	We analysed all available data. The sample sizes of viral genomes was restricted by the amount of publically available data (>1million sequences). Sample sizes for secondary data from published sources (antibody pharmacokinetics, vaccine efficacy data, DMS data) were determined in the original studies: Antibody pharmacokinetics Refs: 19,47-53; DMS: Refs. 4-5,17-18; vaccine efficacy data as stated in Supplementary Tables 4-5.
Data exclusions	No data were excluded from the analyses. For viral genetics data in Germany we used all 'randomly sampled' data available within the time window of interest. For all other countries this information was not available to us through GISAID.
Replication	The reproducibility of the findings was confirmed by independent execution of the computational codes (which are purely deterministic).
Randomization	We did not form any sub-groups of data.
Blinding	We did not perform blinding.

Reporting for specific materials, systems and methods

We require information from authors about some types of materials, experimental systems and methods used in many studies. Here, indicate whether each material, system or method listed is relevant to your study. If you are not sure if a list item applies to your research, read the appropriate section before selecting a response.

Materials & experimental systems

n/a	Involved in the study
☒	☐ Antibodies
☒	☐ Eukaryotic cell lines
☒	☐ Palaeontology and archaeology
☒	☐ Animals and other organisms
☒	☐ Clinical data
☒	☐ Dual use research of concern
☒	☐ Plants

Methods

n/a	Involved in the study
☒	☐ ChIP-seq
☒	☐ Flow cytometry
☒	☐ MRI-based neuroimaging

Plants

Seed stocks	N/A. Does not apply
Novel plant genotypes	N/A. Does not apply
Authentication	N/A. Does not apply
